# Exosomes as cell-derivative carriers in the diagnosis and treatment of central nervous system diseases

**DOI:** 10.1007/s13346-021-01026-0

**Published:** 2021-08-07

**Authors:** Gayatri Gopal Shetgaonkar, Shirleen Miriam Marques, Cleona E. M. DCruz, R. J. A. Vibhavari, Lalit Kumar, Rupesh Kalidas Shirodkar

**Affiliations:** 1 Department of Pharmaceutics, Manipal College of Pharmaceutical Sciences, Manipal Academy of Higher Education, 576 104 Udupi, Karnataka India; 2 Department of Pharmaceutics, Goa College of Pharmacy, 18th June road, Panaji 403 001 Goa, India; 3 Department of Pharmacology, Manipal College of Pharmaceutical Sciences, Manipal Academy of Higher Education, 576 104 Udupi, Karnataka India

**Keywords:** Exosomes, Blood–brain barrier, CNS diseases, Targeted delivery, Diagnosis

## Abstract

Exosomes are extracellular vesicles with the diameter ranging from 50 to 100 nm and are found in different body fluids such as blood, cerebrospinal fluid (CSF), urine and saliva. Like in case of various diseases, based on the parent cells, the content of exosomes (protein, mRNA, miRNA, DNA, lipids and metabolites) varies and thus can be utilized as potential biomarker for diagnosis and prognosis of the brain diseases. Furthermore, utilizing the natural potential exosomes to cross the blood–brain barrier and by specifically decorating it with the ligand as per the desired brain sites therapeutics can be delivered to brain parenchyma. This review article conveys the importance of exosomes and their use in the treatment and diagnosis of brain/central nervous system diseases.

**Graphical abstract:**

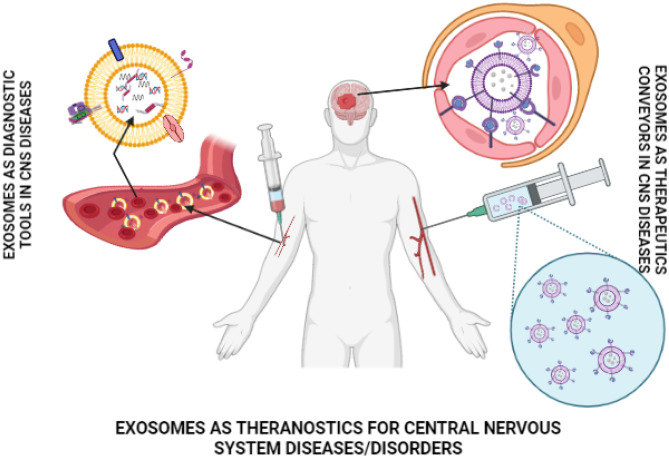

## Introduction

Brain neurological disorder/disease is one of the major causes of disability and death worldwide. It is the second leading cause of death as per 2016 estimate and is the leading cause of disability adjusted life years [1]. As per the study conducted, it was interpreted that globally, the burden of neurological disorders will continue to increase as per absolute number of disability-adjusted life years (DALYs). As population is growing and aging, the prevalence of the disease is increasing with the age, wherein government will face increasing demand for treatment, rehabilitation and support services. Thus, vast research is directed towards prevention and therapeutic methods for treating brain disease. This study was reported for neurological disorders like brain and other central nervous system (CNS) cancers, traumatic brain injury, encephalitis, meningitis, stroke, Alzheimer’s disease and other dementias, Parkinson’s diseases, multiple sclerosis, motor neuron diseases, epilepsy, migraine, headache and other common neurological diseases. The cost associated with brain disease is immense and expected to increase exponentially [1]. Safe and effective treatment of neurological disease/disorder is limited because of poor entry of drugs into the brain parenchyma. This is due to enzymatic degradation, blood–brain barrier, short circulation lifetimes and reduced tissue penetration and so on. Thus, overcoming these problems has become one of the most significant challenge in brain diseases therapy. Nanotechnology has the potential for addressing these challenges which can be further resolved through biological, physical or chemical modification strategies which help in controlled delivery of drugs, imaging and gene modification. Nanoparticle is a helpful mean to improve delivery efficacy, increase the therapeutic effect and reduce the off targeted effects and side effects. The research progress of nanoplatform for brain disease diagnosis and intervention uses the routes such as intravenous, intranasal and local such as intracranial injections.

**Fig. 1:**
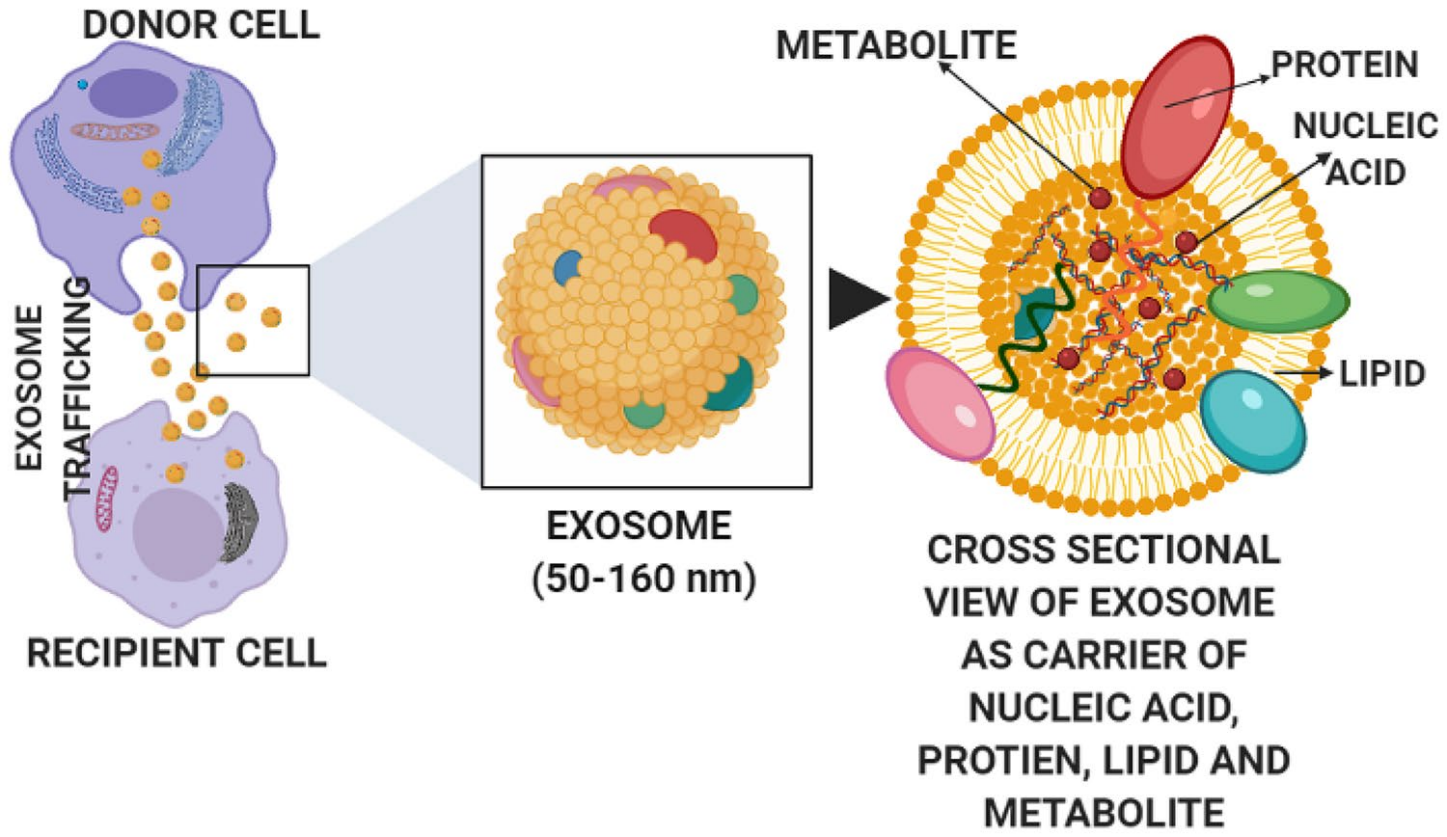
Exosome trafficking between cells

**Fig. 2:**
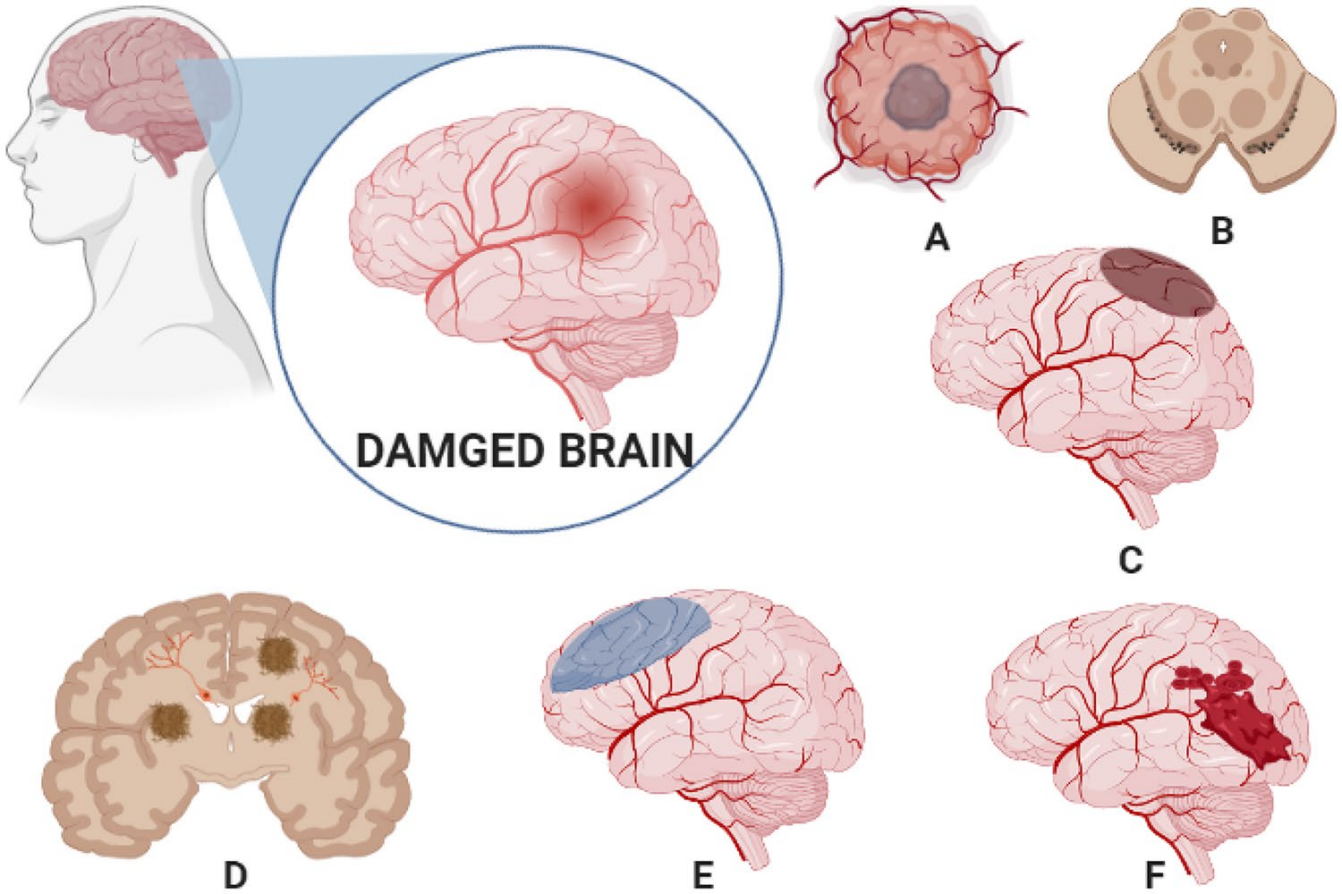
Various brain diseases treated using exosomes. **A** Glioma, **B** Parkinson’s, **C** brain stroke, **D** Alzheimer’s diseases, **E** brain ischemia, **F** brain haemorrhage

Extracellular vesicles are the natural nanocarriers that pack bioactive molecules such as proteins and coding and non-coding RNAs that help in transferring information between cells and tissues and thus playing essential role in cellular communication [2]. They are broadly classified into three types based on the size and morphology, namely, macrovesicles or microparticles, the apoptotic bodies and exosomes. The macrovesicles are obtained from plasma membrane and have diameter ranging from 100 to 1000 nm. The apoptotic bodies are obtained from apoptotic bodies or dying cells that ranges from 1000 to 5000 nm. Exosomes are the smallest form of extracellular vesicles that range from 50 to 100 nm [3]. Besides these three main subtypes, other extracellular vesicles include membrane particles, exosome-like vesicles, neutrophil originating extracellular vesicles (ectosome) [4], prostate originating extracellular vesicles (prostasomes) [5], migrasomes [6], oncosomes, large oncosomes [7], tolerosomes (from intestinal epithelial cells) [8], gesicles [9] and prominosomes (luminal fluid of embryonic neural tube) [10]. Exosomes were discovered in 1983 when it was found that reticulocyte releases 50 nm small vesicles that carry transferrin receptors into the extracellular space [11, 12]. It is the subgroup of extracellular vesicles that released from almost all types of cells. These are cup shaped (Sac-like structures) in morphology, bilayered and spherical in structure under electron microscope [13]. They are isolated from several kind of extracellular liquids such as serum, urine, amniotic fluid, saliva, menstrual blood, cerebrospinal fluid (CSF), serous cavity effusion, or breast feed and ascites [14]. Their dual layered membrane and nanosize protect their load from removal from the body by macrophages, thus enhancing their existence and improving biotic function. Recently, increasing evidence has demonstrated that exosomes have emerged as promising carriers in brain diseases, thus making them potential diagnostic and therapeutic agents (Fig. 1). Figure 2 represents the studies done on various brain diseases treated using exosomes. Recent biodistribution studies of unmodified exosomes after IV administration revealed a rapid accumulation of exosomes in organs of reticuloendothelial system (RES) and also showed few exosomes delivered in the brain; hence, targeting characteristics require improvement before exosomes can be used to deliver brain therapies and is improved by surface modification [15].

## Biogenesis of exosomes

The extracellular biogenesis involves:

1. Formation of early endosome from the plasma membrane through a process of inward budding.
2. Formation of late endosome, microvesicles containing intraluminal vesicles (ILVs).
3. On fusion of late multivesicular bodies (MVBs) with a plasma membrane, the content is released and, if it fuses with lysosomes, it is degraded.

Endosomal sorting complex required for transport (ESCRT) such as ESCRT-0, I, II, III and accessory proteins is involved in biogenesis, formation and vesicle scission [16–18]. Ubiquitin fusing subcomponent of ESCRT 0 starts ESCRT machinery by identifying and sorting ubiquitinated protein to precise endosomal film areas. ESCRT III a protein composite that is concerned with bud formation is activated once ESCRT I interfaces with ESCRT II complex. Finally, the protein IV associated with Vacuolar protein sorting provides energy, followed by the split-out growth to form the ILVs and the ESCRT III compound split up from the MVB film [18, 19]. Another mechanism other than ESCRT has also been introduced that looks close to the mechanism used by budding viruses, wherein exosome biogenesis linked to membrane invagination continued by phospholipid, such as lysobisphosphatidic acid and ceramide, tetraspanin and protein such as heat shock proteins [18, 20–24]. Exosome biogenesis independent of ESCRT machinery is mediated by sphingolipid ceramide, guanosine triphosphate–binding protein (GTPase), ADP-ribosylation factor 6 (ARF6) and its effector phospholipase D2 in cells like melanocytes [14, 21, 25]. Endosomal secretion is regulated by the Rab family (Ras superfamily of small G proteins), specifically Rab 27A, Rab 27B, Rab 11, Rab 35 and Rab 7 [26–28]. The maturation and secretion of exosomes in some cells such as haemopoietic stem cells and leukaemia inducing cells are regulated by vacuolar protein sorting protein 33b [29]. Soluble N-ethylmaleimide sensitive factor attachment protein receptor complex (SNARE) and the pH of the microenvironment are also involved in the fusion between plasma membrane MVBs, and thereby, exosomes release is controlled [30–32]. The ExoCarta database lists thousands of exosome cargo candidate proteins and RNAs, but these cargos vary among various cell lines (http://www.exocarta.org). Most of the studies mentioned that the process by which exosomes selectively pack their cargos remains uncertain.

## Exosome uptake by recipient cells and exosome cargo

The process of exosome trafficking involves the release of exosome in the extracellular space followed by its uptake by the recipient cells, the mechanism of which is still not well known and it remains uncertain. In some of the cellular responses generated by exosomes, it does not require exosome uptake and it is produced by exosome surface protein adhesion to the receptors on cells such as Fas ligand (FasL) or TRAIL (tumour necrosis factor–related apoptosis-inducing ligand) on exosome membrane present on the recipient cells. In the case of ribonucleic acid (RNA) transfer, exosome uptake by recipient cell is important compared to transfer through membrane. Macropinocytosis or phagocytosis, receptor-mediated endocytosis and fusion are different methods of cellular exosome absorption. Exosome absorption is not automatic but relies on the interaction between exosome proteins and recipient cells on the surface. Numerous studies have indicated that exosome surface adhesion–related molecules such as tetraspanin, glycoproteins and integrins decide which cell receives exosome [33].

## Advantages of exosomes as brain-targeted carriers

1. Exosomes are natural nanoscale transport vesicles of messenger RNA (mRNAs), microRNAs and proteins, receptors and enzymes and thus play an important role in theranostics of brain diseases [34].
2. This sort of vesicles (40–200 nm) is secreted by several natural cells, and thus, production becomes easy [35].
3. Due to the existence of major histocompatibility complex (MHC) molecules and a co-stimulatory cluster of differentiation 86 (CD86) molecule on the surface, these vesicles display low immunogenicity, which can potentially improve immune response, especially with chronic exposure and excellent biocompatibility [35].
4. Exosomes have demonstrated inherent stability [36].
5. There may be intrinsic therapeutic benefit in certain unmodified exosomes [37].
6. Drug delivery efficiency is very high [36] and has a high loading capacity for cargo and cargo safety [38].
7. In several drug delivery investigations, it has been exploited as a drug transporter based on which novel therapeutics can be loaded for treatment of brain ailments [39].
8. The exosome-based approach requires a simple, efficient and accurate biosynthesis and self-assembly mechanism [39].
9. Another comparison to the advanced and poorly regulated synthesis methods used to integrate peptides and antibodies into targeted vehicles is that exosomes can be genetically engineered to increase delivery capability and target specificity, thus specifically targeting drugs to the brain [39].
10. Microvesicles (MVs) can also be configured to express particular ligands on the surface of the membrane; then, they can penetrate through the specific tissues with artificially engineered MVs [40].
11. Exosomes have been reported to be involved in many cellular functions due to their release from many cell types and involvement in biological fluids, including protein secretion, immune response control, antigen presentation, RNA and protein transfer, infectious cargo transmission and cell–cell signalling based on these properties exosomes paves good path for brain targeting [41].
12. Exosomes can operate at a near range and even at a distance through the transfer of biological fluids such as plasma [37].
13. As potential diagnostic tools, exosomes also hold great promise as they are released by tumour cells and contain tumour protein biomarkers such as the epidermal growth factor (EGF) receptor variant in glioblastoma [42].
14. The lack of unnecessary accumulation of therapeutic cargo in the liver and the low homing of exosomes to the liver could explain the favourable toxicity profile as well as the high efficiency of delivery to the brain by these vesicles is the major advantage of the use of exosomes compared to other nanoparticle delivery vehicles [43].
15. Exosomes are relatively stable in the blood as they avoid opsonin’s, coagulation factors [44] and the existence of CD 47 on the exosome surface known as the “do not eat me” signal allows them to avoid macrophages [45].
16. The small exosome size should also be helpful in preventing particles from phagocytosis by the mononuclear phagocyte system (which clears particles > 100 nm in size), bypassing lysozyme swallowing and promoting their extravasation by vessel fenestration and passage through the extracellular matrix [38].
17. The discovery that exosomes bear parent cell–derived cargoes gave rise to the idea that they could provide tissue-specific disease biomarkers, and the diagnostic value of exosome cargoes in cancer, heart disease, infection and pregnancy is now being thoroughly explored [38]. As such, exosomes may have pleiotropic biological roles, including vascular homeostasis modulation and control, presentation of antigen to T cells, angiogenesis, transport of cytokines, transmission of reactive oxygen species and transfer of de novo translation mRNA to the recipient cell.
18. Drug delivery vehicles derived from exosomes have wider distribution of biological fluids, likely to produce longer circulation time and potentially improved efficacy [46].
19. Research shows that exosomes derived from mesenchymal stem cells (MSC-exo) retain some of the features of their parent MSCs, such as immune system control, neurite outgrowth regulation, angiogenesis promotion and the ability to regenerate damaged tissue, such as after kidney injury [47].
20. Loading or expressing a therapeutic agent in or on exosomes extends its half-life, achieving impressive effectiveness by delivering the drugs to the intended target [48].
21. Repeated systemic exosome administration does not cause liver toxicity, which supports its safety profile [49].

## Isolation and purification methods for exosome in brain diseases

Researchers are exploring advanced methods for exosome isolation and purification considering its potential to be used as biomarkers and therapeutics. It is isolated by various methods using its physical properties such as size, float density and marker protein presence such as Alix, tumour susceptibility gene (TSG 101), heat shock protein (HSP 70) and CD9 [50] surface receptors from cell culture medium and amniotic fluid, urine, breastfeed and blood. Major studies that reported on brain-targeted exosomes focus on centrifugation and size exclusion chromatography as isolation and purification methods. Different methods of exosome isolations are as follows:

1. Differential ultracentrifugation and density gradient centrifugation: It is regarded as the gold standard method of exosome isolation. It involves the application of centrifugal force to an exosome-containing solution (cell culture media or biological fluids) [51], for example, isolation of astrocyte-derived exosomes to carry microRNA-17-5p to protect neonatal rats from hypoxic ischemic brain damage by inhibiting Bcl-2 and nineteen kilodalton-interacting proteins 2 (BNIP 2) expression [52]. Another study also reports the use of ultracentrifugation technique for isolation of exosome to entrap curcumin for treatment of ischemic brain [53].
2. Immunoaffinity chromatography: In this technique, antibodies are covalently bonded with beads, filters or other matrices, wherein it will bind to the surface protein or antigen present on the targeted exosomes (buffer is used to collect bound fraction from the stationary phase). The non-targeted exosomes will remain free. It isolates exosomes in pure form as their isolation depends on antibodies recognition [51]. Immunoaffinity-based methods such as enzyme-linked immunosorbent assay (ELISA), immunomagnetic nanotechnology or diamagnetic beads are all coated with exosome-specific antibodies and combination chemistry for exosome capture [54].
3. Size exclusion chromatography: In this, different size components are separated based on their size. This method utilizes heterosporous beads packed columns and the elusion time, which is inversely related to the particle size. It helps in maintaining integrity of the exosomes [51]. For example, exosomes were isolated using exo Easy maxi kit from 293 T cells for the treatment of glioblastoma by entrapping miRNA-21 antisense oligonucleotide [48]. Exosomes were isolated from tumour cell line to cross the blood–brain barrier [55]. Similarly, exosome isolation kit was used (EX01-8) in a study on metformin that increased exosome biogenesis and secretion in U87MG human glioblastoma cells: a possible mechanism of therapeutic resistance [56]. Patented and commercialized products like qEV (IZON), exopure™ (bio vision) and exo-spin™ columns (CELL guidance system) have been used in studies for the isolation [54].
4. Polymer precipitation: In this technique, a solution of PEG (8000 Da) is mixed with bio fluid containing exosomes by incubating overnight at 4 °C. Then, composition is centrifuged. Furthermore, it utilizes pre-isolation step (centrifugation) to remove lipoprotein as contaminant and post-isolation step (SephadexG-25 column) to remove polymer [51]. Other precipitating reagents like total exosome isolation reagent for serum, plasma and cell culture media (Invitrogen), 101Bio (Fisher Scientific), Mag Captre™ exosome isolation kit PS (Fuji Film Wako Pure Chemical Corporation), exosome purification and RNA isolation kit (Norgen Botech Corp,) have been developed for exosome isolation with quick and easy step [54]. For example, exosomes isolated using isolation reagent (Invitrogen) in a study on exosomes contribute to transmission of anti-human immunodeficiency virus (HIV) activity from toll-like receptor-3 (TLR3)–activated brain microvascular endothelial cells to macrophages [57]. In another study on differential serum exosome microRNA profiling in a stress induced depression rat model, Invitrogen was used [58]. Polyethylene glycol (PEG) was also used in a study to isolate the exosome from saliva and to study their protein cargo in the progression of cognitive impairment in Alzheimer’s diseases [59].
5. Microfluidic technology: It is based on immunoaffinity, sieving and trapping exosomes on porous structures. This approach requires a smaller starting material volume and minimal processing time for highly pure exosome preparation [51].
6. Ultrafiltration: It is a membrane separation technique based on size and molecular weight of membrane used for separation of exosomes [60].
7. Magnetic separation: The capture and separation of exosomes includes antibody-modified magnetic beads. Due to its contactless, high through performance and precise separation, this method is often used. The exosomes are preserved by immunomagnetic beads while the phosphate buffer washes away other contents. In the chamber, these beads are further lysed, captured and analysed [60], for example, isolation of exosome brain-derived neurotropic factor (BDNF) that utilizes magnetic beads (protein G) modified using BDNF-specific antibodies in a study that reports macrophage exosome for protein delivery to the inflamed brain [61].
8. Acoustic fluid separation: It utilizes the principle of separation based on size. In acoustics, depending on the size, particles are subjected to various acoustic forces and thus differentiating. It is a label free and contactless process, and thus, it is validated and then used for separation [60].
9. Dielectrophoretic separation (DEP): It operates on the theory of a non-uniform electric field generated dielectric forces experienced by polarized particles. Cell’s intrinsic dielectric properties, size of the particles, magnitude and frequency of electric field determines the magnitude of forces exerted by DEP on exosomes. The larger polarized particles attract towards lower electric field while exosomes are attracted towards higher electric [60].
10. Deterministic lateral displacement (DLD) separation: It uses devices or the chips and is based on the principle of particle flow path which is greater than the critical size. Researcher faces difficulties with separation and clogging (it is easy to use and label free) [60].
11. Nanotrapped wire: In this process, a polymer nanoporous membrane is used or porous structures are used to trap exosomes, such as nanowires (silicon based) arranged with micropillars. This technique operates on the concept of exosomes trapping based on the size. Degradation of silicon leads to generation of silicic acid [18].

## Loading of exosomes

Exosomes have a liposome like membranous structure that motivates researchers to extend their prior drug loading experience in them [45]. The loading strategy may be a crucial factor in understanding of commercial and clinical use of nanocarriers. For biological applications, many studies identify different methods of exosomes encapsulation [18]. 

### Preloading of exosomes

Preloading of exosome is a common technique for achieving desired target specific exosome which is accomplished before exosome formation and isolation by treating or transfecting cells.

This is a common technique for specifically targeting exosomes that is achieved before exosome formation and isolation by treating or transfecting cells [18]. This technique is based on two methods as given below:

#### Incubation of cells with cargo

This process utilizes hydrophilic or hydrophobic drug or salt solution for incubation of parent cell for the desired time, and the cells will exocytose these substances in the form of loaded vesicles. Packing of these exosomes is depending on interaction between materials and cells [18].

#### Gene transfection

In this method, parent cells are manipulated using commercialized transfecting reagents with MicroRNA (miRNA) or small-interfering RNA (siRNA) or plasmid (pDNA) that the cells load into the inner core of the extracellular vesicles (EVs) or stack for excretion on the outer layer using therapeutic application [62–66]. A further mechanism for cell transfection is metabolic labelling. In this case, synthetic metabolites such as oligonucleotides, lipids, glycans or amino acids are supplemented with cell culture medium to biosynthesis genes, lipids, glycomes or proteomes, respectively [18, 67–69].

### Post-loading of exosomes

Exosomes are non-living structures hence substances and reaction conditions are feasible for surface functionalization. Additionally, during cell-based modification only limited amount of content envelops inside the vesicles. Exosomes are isolated and purified using ordinary liquids such as culture medium, serum or breast feed for further processing during post-loading [18]. Post-loading methods are given below:

#### Incubation

The method of incubation is used for exosome loading by incubating them with cargo of interest, especially hydrophobic interfaces. After incubation with purified exosomes, many small lipophilic drugs, such as curcumin, dopamine, celastrol, porphyrin, cucurbitacin, methotrexate and doxorubicin, were competently stacked at room temperature [18, 35, 70–75].

#### Sonication and extrusion method

This technique has been developed to expand packaging quality. Sound energy as an automatic force is used in sonication to interrupt the exosome layer so that cargo or theranostic agents can be loaded in them. Shear mechanical power is used as a lipid extruder to connect exosome and agents under controlled temperature in the extrusion technique [18].

#### Antibody-specific loading

Exosomes contain the genomic and proteomic material of their originating cells, whereas on the outside of exosomes, definite antibodies can be bound to a particular antigen called antibody-specific loading. However, through gene transfection, non-native receptors could be added to the exosomes [18].

#### Electroporation

In order to improve the layer permeability and loading of hydrophilic agents, including miRNAs, siRNAs, smaller drugs and electroporation of superparamagnetic iron oxide nanoparticles (SPIONs), it is often possible to use the approach to make pores on the exosome lipid dual layer film by applying an electrical field (150–700 V) to allow the loads to be stacked inside the exosomes [34, 43, 73, 76–78]. Furthermore, colloidal stability of exosomes is improved using membrane stabilisers [18, 79].

### Freeze and thaw method

Due to the aggregation of sphingomyelin, cholesterol and gangliocytes, exosome shows more rigid lipid bilayer in comparison to the cell membrane. Exosome can be engineered by incubating at room temperature for fixed time with therapeutic agent followed by repeated freeze and thaw period (freeze at − 196 °C and thawed at or higher room temperature, i.e. 40 °C) [18, 31].

#### Saponin-assisted method

It can have a higher internalization level. Saponin is an active compound that forms complex with cholesterol on the exosome surface and creates holes followed by improving membrane permeability [18, 80].

## Characterization of exosomes

Based on size, structure, protein and lipid content, exosomes can be characterized [51]. This helps to understand exosomal properties and activities as they affect the loading and delivery of drugs. In 2014, the International Society for Extracellular Vesicles published a paper proposing the classification of exosomes by the inclusion of exosome-associated surface markers and the absence of protein that is not exosome-associated. Dynamic light scattering and nanoparticle tracking analysis are known as optical methods for traditional exosome detection techniques, whereas non-optical methods include transmission electron microscopy (TEM), atomic force microscopy and enzyme-linked immune sorbent assay. These method’s drawbacks include complicated machinery, poor sensitivity and high use of reagents. However, we can achieve high performance with high precision and low reagent consumption by microfluidic detection technique. These include fluorescence correlation microscopy, colorimetric detection, surface Plasmon resonance detection and nuclear magnetic resonance detection [60].

## Exosomes and mechanisms to cross blood–brain barrier

The ability to bypass blood–brain barriers (BBB) is one of the most important core features of exosomes in the treatment of brain diseases or disorders, representing a promising strategy for the treatment of brain diseases [49]. Approximately 98% of the drug shows disadvantage of not crossing BBB, which is overcome by nanoformulations. These, however, show nanotoxicity and rapid drug clearance. To solve this problem, polyethylene glycol (PEG) is used, but the delivery of drugs to the brain and the interaction between target cells is decreased. Using exosomes as a drug delivery system, this kind of complications can be controlled as it is the natural product of the body and can be personalized to cross the blood–brain barrier and increase the distribution of drugs to the brain by decreasing the mononuclear phagocytic drug clearance. However, the exact mechanism of interaction between exosomes and BBB is poorly understood. Some researchers have proposed the mechanisms as follows:

1. Transcellular route: Exosomes are internalized by endothelial cells of BBB through cell type–specific protein via receptor-mediated endocytosis and then undergoes transcytosis.

2. Paracellular route: Exosome cross the intercellular junction of endothelial cells of BBB and then enter the central nervous system (CNS).

Evidence showed that Parkinson’s disease was treated by using antioxidant protein catalase loaded exosomes by crossing blood–brain barrier after intranasal administration [81]. In cross-talking between neurons, astrocytes, microglia and oligodendrocytes, the release of EVs has been recognized as an essential modulator, not only in the physiology of the brain but also in the neurodegenerative and neuroinflammatory diseases. EVs also contribute to the intercellular communication in the brain through their basal release and uptake by surrounding cells or release into the CSF and blood, in addition to direct, paracrine, endocrine and synaptic cell–cell associations. Their cargos make them the potential source of biomarkers. In a study conducted to establish how exosomes bypass the BBB, the author demonstrated that exosomes carrying luciferase which is able to cross the brain microvascular endothelial cells (BMEC) monolayer in the inflammatory condition but not in the usual condition in a study of interaction between exosomes and BMECs. Most exosomes have also been shown to bypass the BMEC monolayer via the transcellular path followed by endocytosis, multivesicular body (MVB) formation and exocytosis steps in determining the paracellular pathway [49, 82]. Thus, exosome which bypasses or penetrates the BBB is required a successful delivery of therapeutic/diagnostics agent in the brain [83]. To overcome the problems associated with BBB, exosomes were modified with brain homing peptides which targets brain endothelium. However, naive macrophage–derived exosomes and its surface proteins can also be used for CNS targeting, such as integrin lymphocyte function-associated antigen 1 (LFA-1), intercellular adhesion molecule 1 (ICAM 1) and carbohydrate-binding C-type lectin receptors, to interact with brain microvessels endothelial cells [61]. Pathology of many brain neurological disorders such as multiple sclerosis, Alzheimer’s disease, Parkinson’s disease, stroke, brain tumours, traumatic brain injuries and others can result in BBB dysfunction. Based on this, a study was reported utilizing natural macrophages exosomes to cross the BBB that utilizes the integrin lymphocyte function associated antigen 1, intercellular adhesion molecule 1 and the carbohydrate binding C type lectin receptors to interact with brain microvessel endothelial cell that comprises the BBB [61]. Brain capillary endothelial cells express transferrin receptors (TfR) on its surface; thus, decorating exosomes with T7 as a ligand helps in penetrating exosomes through BBB via the process of transcytosis [48]. Rabies virus glycoprotein (RVG)–modified mesenchymal cell–derived exosomes interact with the acetylcholine receptors present on the surface of endothelial cells of BBB that helps in targeting most of the genetic materials as well as therapeutic agents to the brain [84]. Mesenchymal-derived and immune cell–derived exosomes can efficiently cross the BBB without surface modification. Exosomes derived from hypoxic glioblastoma cell line U87 promotes the proliferation of brain microvascular endothelial cells and enhances the permeability through vascular endothelial growth factor-A (VEGF-A) by reducing the expression of claudin-5 and occludin [85]. Exosome inheriting lymphocyte function-associated antigen 1 from the macrophages, a protein that interact with endothelial intercellular adhesion molecule 1 which mediates lateral migration and diapedesis of exosomes across BBB as mention above [86]. Also, macrophage-derived exosomes depend on their inflammation related targeting properties to cross the BBB and carry the therapeutic agent into ischemic region with or without minimal modification. Exosomes possessing specific surface protein such as integrin can penetrate the BBB and efficiently target tumour tissues. There is report on successful crossing of BBB by exosome modified with neuropilin-1 targeted peptide arginine-glycine-glutamic acid (RGE) through click chemistry for treatment of glioma [47]. Similarly, the arginyl-glycyl-aspartic acid (RGD) conjugated curcumin-loaded exosomes for the treatment of ischemic brain are been reported. However, there is no exact mechanism mentioned for the same [36].

Further research showed that tetraspanin CD9 on the surface of exosomes interacts with surface glycoprotein on target cells and facilitates exosome to fuse with the cell membrane, thus helping in direct cytosolic delivery of gene [87]. It has also been proposed that exosomes can be internalized into MVBs of recipient cells and then release again to be re-internalized into MVBs of secondary recipient cells. Therefore, by moving from cell to cell via the MVB compartment, exosomes can cross multiple layers of BBB [43, 88]. It is reported that metastatic breast cancer secreted exosomes destroy vascular endothelial barrier to promote metastasis. Exosome associated miR-105 significantly down regulate expression of Zonula Occludens-1 (ZO-1) a central molecular component of tight junctions destroying barrier function in endothelial monolayers [89]. Furthermore, the next mechanism reported showed that exosomal miR-181c downregulated expression of 3-phosphoinositide-dependent protein kinase-1 leading to decrease level of phosphorylated cofilin and abnormal polymerization of actin in brain endothelial cells [90]. It has also been reported that brain endothelium–derived exosomes help in crossing the BBB, because of high level of CD63 [91], e.g. VEGF siRNA loaded into the exosomes isolated from the brain endothelium bEND.3 cell culture medium and found that exosomes siRNA could cross BBB to effectively deliver siRNA causing inhibition of xenograft cancer cell aggregation [91]. Furthermore, the features of exosome including their very small size and cell membrane like structure allow them to cross BBB. A study is reported wherein the advantage of increase expression of TfR receptors on the BBB due to stroke is been utilized to transfer enkephalin in the brain using exosomes by crossing the BBB [92]. Folate-decorated exosomes can also pave way for it into the brain through BBB by the process of endocytosis by binding with the folate receptors expressed on the BBB.

## Exosomes in diagnosis and treatment of CNS diseases

Exosomes are shed by the cells under both normal and pathological conditions. They carry nucleic acid, proteins, lipids, metabolites and antibodies from their host cells which indicate the pathophysiological conditions and thus are widely considered to be important biomarkers for clinical diagnostics [93]. These are attractive target for diagnosis, because their content is altered during disease conditions. It can be easily isolated non-invasively from accessible biological fluids like urine, blood and saliva which help in early diagnosis of disease including CNS diseases. Membrane structure of exosomes entrapping its content gives advantage over conventional specimen, since these biomarkers are protected from degradation. Exosomes are highly stable, and hence, drug-loaded exosomes can be stored for prolong time before analysis. It can be traced to its origin as its surface expresses the markers related to its cellular origin. These exosomes can pass through the BBB and thus provide information about CNS cells that are difficult to obtain without invasive techniques [94]. Exosomes can be derived either from plasma or from CSF for the diagnostic purposes of CNS diseases. The presence of particular molecules in exosomes obtained from disease helps to distinguish them from other diseases, such as exosomes derived from B cell lymphoma, which express B cell–specific antigen such as CD19 and CD20, but not expresses in glioma cells. In a study conducted to validate the potential of exosomes content to be used as a biomarker relative to the content of CSF. Exosomes with Aβ92, T-tau and P-T181-tau have the same ability to diagnose Alzheimer’s disease with CSF content and amnestic moderate cognitive impairment, thereby making it an alternative to CSF or PET (positron emission tomography) scans or combining it with CSF biomarkers to boost Alzheimer’s diagnostic potential. The presence of miRNA in exosomes collected from the periphery can also be used as a tool for the same diagnosis. Cerebrospinal fluid collected from exosomes of Alzheimer’s diseases patients showed alpha terminal fragments of amyloid precursor protein C that are used as a diagnostic tool. Dendritic cells derived from multiple sclerosis exosomes isolated from interferon gamma-stimulated red bone marrow produce miRNA-219, which promotes myelination [95]. Furthermore, for the diagnosis of the multiple sclerosis same, proteins present in the exosomes which are tau proteins found in the elevated stage in chronic traumatic encephalopathy (seen in the athletes) can be used as well as RNAs entrapped in exosome neuronal cells can also be regarded as potential biomarkers, especially non-coding RNAs like RP11462422.1. In patients suffering from dementia, serum-derived exosomes reported a decline in the experimental level of miR-223, miR-137 and miR-155 [96]. In diagnosing seizure intensity, miR-4668-5p, miR-3613-5p, miR-197-5p, miR-8071, miR-6781-5p and miR-4322 entrapping exosomes have also been found to be helpful [97].

**Fig. 3:**
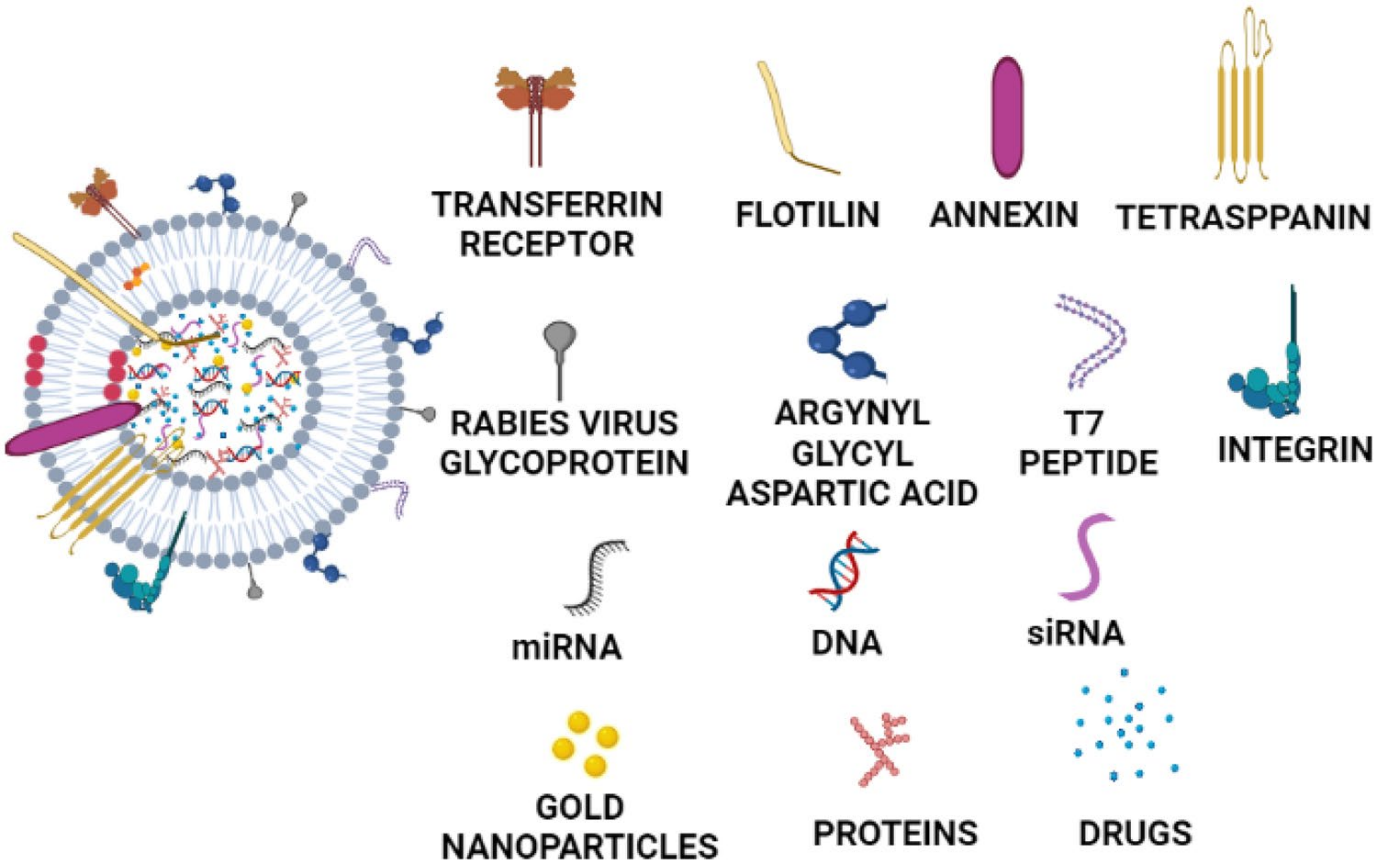
Various cargo-loaded exosomes for treatment of CNS diseases and surface modification of same with ligands for brain targeting

Present therapeutic methods take advantage of unmodified extracellular vesicles bearing beneficial intrinsic properties for the treatment of Alzheimer’s diseases, such as stem cell–derived exosomes. Lipids, proteins and nucleic acid are loaded during exosome development. Sphingomyelin, cholesterol, phosphatidylserine, de-saturated lipids, ganglioside monosialodihexosylganglioside 3 (GM3) and ceramides are part of a lipid. The proteins distributed throughout the exosome membrane or cytostome includes enzyme linked to the formation of fusion proteins, chaperones and MVBs, such as CD9, CD63, CD81, Alix and TSG101. Antibodies, metabolites, mRNAs, miRNAs and other coding as well as non-coding RNAs and DNAs are found in the nucleic acid. Exosome derived from neural cells houses about 6000 known proteins and more than 85% of known miRNAs. Furthermore, the content of exosome is dependent on the type and state of parent cells [33, 98, 99]. Considering these findings, many studies are reported for diagnosis as well as treatment of diseases/disorders: for example, dopamine-loaded blood exosomes for treatment of Parkinson’s diseases [35], nerve growth factor–loaded exosomes derived from human embryonic kidney 293 cells for the treatment of cerebral ischemia [66], curcumin-loaded mesenchymal stromal cell–derived exosomes for the treatment of ischemic brain [36], exosomes enveloped adeno-associated virus vector (vexosome) for gene delivery to the brain [100], miR-219-enriched exosomes from serum for the treatment of multiple sclerosis [95], exosomes loaded with nanoformulated catalase for the treatment of Parkinson’s diseases [81], curcumin-loaded embryonic stem cell exosomes for the ischemia–reperfusion injury [101], siRNA-loaded glioblastoma-derived exosomes for the Huntington diseases [102], siRNA-loaded exosomes for the treatment of Alzheimer’s diseases [103], microRNA-loaded serum-derived exosomes for prediction of different stages of multiple sclerosis [104], miRNA-loaded exosomes for detection of Alzheimer’s diseases [105], α-synuclein-loaded exosomes for detection of Parkinson’s diseases [106], anti-inflammatory molecules like curcumin-loaded exosomes for the treatment of brain inflammatory diseases [71], paclitaxel-encapsulated exosomes from the brain endothelial cells for inhibition of tumour growth [46], curcumin-loaded exosomes for the treatment of brain inflammation and immune encephalitis [71], adipose-derived MSC exosomes for the treatment of inflammation and brain damage in sepsis syndrome [107], doxorubicin-loaded brain endothelial cells derived exosomes for the treatment of brain cancer [46], macrophage-derived exosomes entrapped brain-derived neurotrophic factors for the therapy of brain inflammation [61], curcumin and superparamagnetic iron oxide nanoparticles (SPIONs) for the therapy and imaging in gliomas [47], glial cell line–derived neurotrophic factors entrapped macrophage exosomes for the treatment of Parkinson’s diseases [108] and MSC-derived A1 exosomes for the treatment of inflammation and prevention of neurogenesis and memory dysfunction in epilepsy [109]. Figure 3 represents various cargo-loaded exosomes for the treatment of CNS diseases and surface modification with ligands for brain targeting. Below given section summarizes the details of exosomes targeted for treatment and diagnosis of various brain diseases.

### Exosomes in the diagnosis and treatment of brain strokes, ischemic brain and brain injury

Globally, injuries to the central nervous system, particularly brain strokes, are the principal cause of death in people, which may otherwise result in long-term impairment. Although, to some extent, medically approved treatment for the brain injury owing to cerebral stroke is available to break up the blood clot by administering tissue plasminogen activator (tPA). A diagnostic marker to predict the austerity of the stroke would be immensely useful. Recent research suggests the potential use of exosomes as biomarkers for identifying patients at risk of haemorrhage or other severe complications following a brain stroke, as well as a possible neuroprotective agent as an approach from the treatment angle. Many studies describe groups of mRNA targets as promising non-invasive biological markers for the diagnosis of stroke [110]. It is known that neuronal exosomal concentrations are inflected by factors such as injury and inflammation [111]. One of the research studies investigated the response of acute ischemic stroke (AIS) on the levels of miRNAs in exosomes. The study consisted of randomly selected acute ischemic stroke patients and comparable non-stroke subjects. The study revealed that, when plasma exosome concentrations were analysed, the concentrations of exosomes of stroke patients were considerably greater than that of non-stroke subjects. This further indicates that the expression levels of exosomal miRNAs, namely miRNA-134, were significantly greater in patients following a stroke in comparison with that of non-stroke subjects [112]. Additionally, it was found that the elevated expression levels of the exosomal miRNA-134 were correlated with poor prognosis of stroke. This data suggested that exosomal miRNAs could be deemed as promising biomarkers for diagnosing AIS, for distinguishing AIS patients from non-stroke patients and help in assessing the extent of impairment owing to the ischemic injury. Another group of researchers reported the associations between levels of miRNAs in serum, severity of stroke and the involvement of miRNAs in exosomes, in the regulation of inflammatory responses following a stroke. A retrospective case–control study was designed to observe the levels of miRNA-223 in test subjects and healthy subjects. RNA was obtained from serum exosomes, and the levels of miRNAs were analysed by the polymerase chain reaction (PCR) test. The study utilized miRNA-16 as an internal control and when tested, the expression levels of miRNA-16 were almost the same in both the test and healthy groups. The expression levels of exosomal miRNA-223 of the test group were elevated in correlation with the healthy group. It was also evident that the stroke patients with poor outcomes were inclined to have greater expression levels of exosomal miRNA-223 [113]. This revealed that elevated miRNA-223 can be linked to the brain strokes and could be of possible diagnostic value in determining the severity of stroke. In 2018, another research group examined the levels of exosomal miRNAs in plasma, in different phases of ischemic stroke (IS) [114]. The study consisted of patients with IS and corresponding non-stroke controls, and the patients were categorized into groups: hyper-acute phase IS (HIS), AIS, subacute phase IS (SIS) and recovery phase IS (RIS). On analysis of the serum exosomal miRNAs, the expression levels of miRNA-21-5p in subacute phase IS and recovery phase IS are being found to be considerably greater than those of controls, while those in AIS were lower than controls. It also revealed that the expression levels of both the miRNAs in AIS were decreased in comparison with the HIS group. Thus, this study indicates that the combination of serum exosomal miRNAs, miRNA-21-5p and miRNA-30a-5p, could be potential biomarkers for diagnosing ischemic stroke, as well as help in characterizing the phase of IS.

Studies propose that the delivery of MSC exosomes has restorative effects in patients with stroke and improves post-stroke neurodegeneration and prevents post-ischemic immunosuppression [115]. In another study, the effect of MSC-derived exosomes was reported and they also compared them with native MSCs, which were administered intravenously to “C57 black 6” mice, following focal cerebral ischemia. The MSC exosomes were delivered on the 1st, 3rd and 5th day after stroke, and MSCs were delivered on day 1 of stroke. After 28 days of stroke and timely experimental MSC/MSC exosome administration, the histological brain injury, motor coordination loss, cerebral neurogenesis and immune responses of the test mice were analysed [116]. The results established that the mice in the study that received MSC exosomes from two different bone marrow–derived MSC lineages showed improvements in neurological damage and long-term neuroprotection. This proved the potential use of MSC exosomes as a treatment for stroke. Present research suggests that the assessment of serum exosomal miRNA-126 could be used to identify severe continual ischemia and aid in differentiating it from mild injury after short-term ischemia. A study investigated the potential of modified exosomes loaded with miRNA-126, to protect against injury from brain ischemia in a rat model of middle cerebral artery occlusion (MCAO). The study utilized adipose-derived stem cell (ADSC) — exosomes loaded with miRNA-126, which was administered to MCAO rats. Upon analysis, it showed that the levels of miRNA-126 were significantly reduced in MCAO rats. Furthermore, cell activity assays and behavioural tests showed that the miRNA-126 exosomes promote neurogenesis and angiogenesis as well as inhibit microglial activation and inflammatory response after stroke [117]. This data indicates that miRNA-126 exosomes have a potential role in regulating neurogenesis and neuroinflammation and could be used as a new approach to treat ischemic stroke.

Hypoxia–ischemia is the primary cause of brain damage in premature and full-term neonates, which is eliciting higher morbidity and mortality rates worldwide. Perinatal hypoxic-ischemic brain injury in preterm new borns has led to long-term neurological complications, and so far, no conclusive therapeutic strategies are available. A study was conducted wherein ovine foetuses with hypoxic-ischemic encephalopathy (HIE) was studied and it demonstrated the neuroprotective properties of MSC-derived exosomes in preterm brain injury. Cerebral hypoxia–ischemia was inflicted upon the ovine foetuses by brief umbilical cord occlusion to mimic the conditions under which hypoxic-ischemic brain injury occurs in neonates. In-utero intravenous MSC exosomes were administered, and its therapeutic efficacy was analysed by determining the changes in structural injury by microscopical examination or biopsy of the brain, evaluating the seizure burden and anti-inflammatory effects. It was reported that the systemic administration of MSC exosomes improved functional recovery, reduced cognitive impairments, induced long-term neuroprotection and stimulated neurogenesis and angiogenesis [118]. It was also shown to lower the extent of seizures, thereby enhancing overall brain function. The study reported a potential therapeutic strategy for cerebral ischemia in neonates, by functional protection of the central nervous system, after administration of MSC exosomes.

The incidence of traumatic brain injuries (TBI) in young adults (15–24 years) and older adults (≥ 75 years) in the world, especially in the United States (US), is expanding exponentially and diagnostic strategies for assessing the degree of neurological damage and to timely prevent complications related to brain injury, as well as to predict the response to therapy are imperative. Because of the inability to biopsy neurological components, injury-linked biological markers are essential to define the pathophysiological mechanisms and predict the neurological outcomes. One such approach to a circulating biomarker is the use of exosomes, i.e. “liquid biopsy”. Several researchers have reported changes in exosomes in the plasma and CSF of patients with TBI. In 2008, a study initially demonstrated the potential of circulating exosomal RNAs as a diagnostic tool for patients with TBI. Furthermore, investigations demonstrated that exosome release after injury mediates the production of pro-inflammatory cytokines and elevations in the interleukin-1 cytokines, especially, interleukin-1α, interleukin-1β and interleukin-18 after a TBI was observed [119]. As documented, interleukin-1β, when released, triggers an inflammatory response and reinforces other bioactive signalling molecules like cytokines and proteases. The levels of interleukin-1β have reported to be raised significantly within 10 days of injury [120]. Studies have also revealed that within 24 h of brain injury, the levels of interleukin-1β increase in systemic circulation and CSF, and these may prove to be possible prognostic determinants as interleukin-1β has found to be highly elevated in subjects with major brain injuries along with heightened intracranial pressures (ICP). It was proposed that neuronal exosomes purify from peripheral blood samples as a diagnostic tool for acute brain injury. Synaptopodin (SYNPO), a cytoskeletal actin–associated protein present in postsynaptic spines, was evaluated as a possible biological marker. They initially hypothesized and later reported that damaged neurons, as in the case of TBI, scavenge synaptopodin from exosomes in order to aid in the repair of cellular damage, and thus, synaptopodin depletion in exosomes is a determinant of neuronal injury [121]. Similar, studies have been reported for neonatal hypoxic-ischemic encephalopathy (HIE) and few clinical studies have reported the use of blood-based exosome biomarkers in brain injury. Another study reported an elevation in the levels of microtubule associated proteins (MAPs) in exosomes, specifically, tau proteins and phosphorylated tau proteins (p-tau) in aged with mild TBI (mTBI) and repetitive TBI (rTBI) linked to war. The study was conducted with veterans in two groups: mTBI and rTBI. They analysed the levels of tau protein, p-tau protein as well as β-amyloid protein in plasma and exosomes, and the results indicated that tau protein and p-tau protein levels in exosomes were raised in case of repetitive TBI in comparison with mild TBI. The data indicated that these protein biomarkers could have a diagnostic potential [122]. It was demonstrated that repetitive mTBIs were associated with elevated levels of a neuronal cytoplasmic protein: neurofilament light chain (NfL). The study consisted of veterans with a history of TBI, and exosomal levels of NfL, tumour necrosis factor-α (TNF-α) and interleukins (IL-6 and IL-10) were analysed [123]. The results revealed that the exosomal TNF-α levels corresponded with the symptoms of post-concussive syndrome (PCS), post-traumatic stress disorder (PTSD) and the total number of mTBIs corresponded with higher exosomal NfL and lower IL-6. The study suggested that repetitive TBIs are associated with elevated levels of exosomal NfL, with highest elevations in patients with PCS and PTSD. Another study proved that TBI induces changes in exosome-associated miRNAs in a rodent controlled cortical impact (CCI) model. Harrison et al. quantified levels of miRNAs in exosomes from the brain tissues of CCI injured male C57 black mice after 7 days. The miRNA isolates were sequenced and later in situ hybridization was performed to analyse the expression of miRNAs in the brain. The expression of miRNA-21, miRNA-146, miRNA-7a and miRNA-7b was found to have increased in the injured hemisphere of the brain compared to the placebo control, while the expression of miRNA-212 was found to have decreased [124]. An earlier report suggests that miRNA-21 holds a neuroprotective role in TBI. It inhibits apoptosis and promotes angiogenesis, and thus, treatment with miRNA-21 helped in rescuing cognitive impairments and relieving brain hydropsy [125]. All these facts suggest that exosome-associated miRNAs could be used as a diagnostic marker, help in disease progression and serve as a biotherapeutic approach for mediation following a brain injury.

### Exosomes in the diagnosis and management of seizures and morphine relapse

Epilepsy is a complex disorder which has many possible clinical presentations and its diagnosis primarily depends on clinical examination and the patient’s medical history. Since the diagnostic strategies for epilepsy are limited, this poses a great challenge and thus, rapid and non-invasive approaches like biomarkers would be of great significance. In a recent study they demonstrated the use of exosomal proteins to study epilepsy. The group originally identified three major proteins, a blood clotting factor viz Christmas factor (F9), an adhesive glycoprotein thrombospondin-1 (THBS1) and an integral membrane protein amyloid-β precursor protein (APP), and analysed their levels in plasma exosomes [126]. The study affirmed that the expression levels of two proteins, i.e. F9 and THBS1, were considerably different. The group reported that, in the case of subjects stricken with epilepsy, their plasma exosomes showed the expression of F9 to be higher than that of the control subjects. Furthermore, the exosomal levels of THBS1 in test subjects were found to be less compared to the control subjects. Thus, they established that the proteins F9 and THBS1 in plasma exosomes could serve as a potential diagnostic marker for epilepsy and associated seizures. Another study recorded that miRNAs in serum exosomes may be responsible to oversee the seizure progression in patients with mesial temporal lobe epilepsy related to hippocampal sclerosis (mTLE-HS) and thus could be used as possible diagnostic biomarkers for the disease. The study consists of 40 test subjects diagnosed with mTLE-HS and corresponding control subjects. The miRNA profiles of exosomes were investigated, and the levels of these miRNAs (miRNA-3613-5p, miRNA-4668-5p, miRNA-8071 and miRNA-197-5p) could be successfully distinguished from those of control subjects [97]. This suggested that assessing the levels of exosomal miRNAs in serum could be used as a new approach to diagnosing mTLE-HS. Recently, the possibility of exosomal miRNAs in the diagnosis of epilepsy along with a comorbidity of depression was suggested and in addition to this, their opportunity as a new and different therapeutic strategy was proposed [127]. Since exosomes containing miRNAs effuse from CSF following a cerebral disease and can be easily extracted from serum, it could have potential as a biomarker. Similarly, another study group reported that three miRNAs; miRNA-146a, miRNA-155 and miRNA-132 hold a vital task in the progression of genetic generalized epilepsies (GGE) and suggested their diagnostic value in GGE [128].

Studies utilizing MSCs as therapy have currently gained significance for various neurological diseases, and one such study reported the therapeutic potential of MSC-derived exosomes in pilocarpine-induced epilepsy models. The study group treated 1–8-week-old C57 black male mice with exosomes derived from pluripotent stem cells, and the results showed that the native exosomes from bone marrow MSCs have robust anti-inflammatory and neuroprotective properties. The treatment alleviated inflammation as well as reduced neuronal loss, helped in normalization of neurogenesis and significantly improved spatial learning in mice by targeting the astrocytic glial cells in the hippocampus [129]. Additionally, in another study, the researchers tested the prospect of administering exosomes for the treatment of status epilepticus. They reported that the intranasal administration of MSC-derived A1 exosomes to pilocarpine-induced epilepsy model mice resulted decrease in neuronal loss, reduced neuroinflammation, unimpaired cognitive functions and memory preservation [109].

The casualties due to drug dependence are steadily on the rise, and the world is facing a devastating health crisis because of drug abuse and overdose. Society is being crushed by the toll of the opioid epidemic. The growing health problem has called on the urgent need to identify potential therapies to combat opioid addiction. A study was conducted to examine the therapeutic potential of exosome delivered small-interfering RNAs (siRNAs) in case of opioid addiction. A researcher made use of a cell penetrating peptide namely rabies virus glycoprotein (RVG) peptide attached to exosomes, to allow them to pass through the BBB efficiently. These RVG-modified exosomes were loaded with opioid receptor Mu (MOR) siRNA. A study group of C57 black male mice was intravenously injected with the modified exosomes, and siRNA levels were assayed in plasma after 6 h. The siRNA, MOR mRNA and protein levels were also assessed in brain tissues after 24 h. Following a morphine-administered relapse, a conditioned place preference (CPP) test was conducted, and it showed that mice treated with RVG exosomes containing MOR-siRNA exhibited behaviours corresponding to restrained drug addiction in contrast with control subjects administered with saline [40]. The results showed that the RVG exosomes transporting MOR-siRNA evidently supressed morphine relapse by downregulation of MOR expression levels in the brain. This established that siRNAs prevent morphine relapse. These studies suggest that RVG-modified exosomes can effectively deliver siRNAs to the brain for the therapy of drug relapse compared to options such as naltrexone and methadone. Table 1 depicts exosomes in treatment of brain strokes, ischemic brain, brain injury and epilepsy.

**Table 1:** Exosomes in treatment of brain strokes, ischemic brain, brain injury and epilepsy

Disease	Objectives of the study	Activity	Outcomes	References
AIS	Examining the effect of AIS on exosomal miRNAs	Expression levels of exosomal miRNAs in stroke and non-stroke patients were analysed	Expression of miRNA-134 was significantly higher in AIS patients	[112]
Ischemic Stroke (IS)	Characterizing the phase of IS depending on the expression of different miRNAs	Expression levels of exosomal miRNAs in patients with HIS, AIS, SIS and RIS were analysed	Expression of miRNA-21-5p in SIS and RIS, and miRNA-30a-5p in HIS was higher than controls	[114]
Cerebral Ischemia	Examining the potential of ADSC-exosomal miRNA as a treatment approach for cerebral ischemia	ADSC-exosomal miRNA was administered to MCAO rats and behavioural tests and cell activity assays were performed	ADSC-exosomal miRNA-126 regulated neurogenesis and neuroinflammation	[117]
Traumatic Brain Injury (TBI)	Examining levels of protein biomarkers in TBI patients	Exosomal levels of tau, p-tau in plasma of veterans with mTBI and rTBI were analysed	Exosomal tau and p-tau levels were elevated in rTBI compared to mTBI	[122]
TBI	Examining levels of NfL in TBI patients	Exosomal levels of NfL, TNF-α and ILs were analysed in veterans with rTBI	rTBIs were associated with elevated levels of exosomal NfL	[123]
Epilepsy	Examining the use of exosomal proteins to study epilepsy	F9, THBS1 and APP levels were analysed in plasma exosomes	Expression of F9 was higher in epilepsy patients and THBS1 was lower in epilepsy patients compared to controls	[126]
Epilepsy	Examining the potential of MSC exosomes as a treatment approach for epilepsy	Pilocarpine-induced epilepsy mice models were treated with MSC exosomes	MSC exosomes have anti-inflammatory and neuroprotective properties	[129]

### Exosomes in the diagnosis and therapy of brain neurodegenerative disorder

**Fig. 4:**
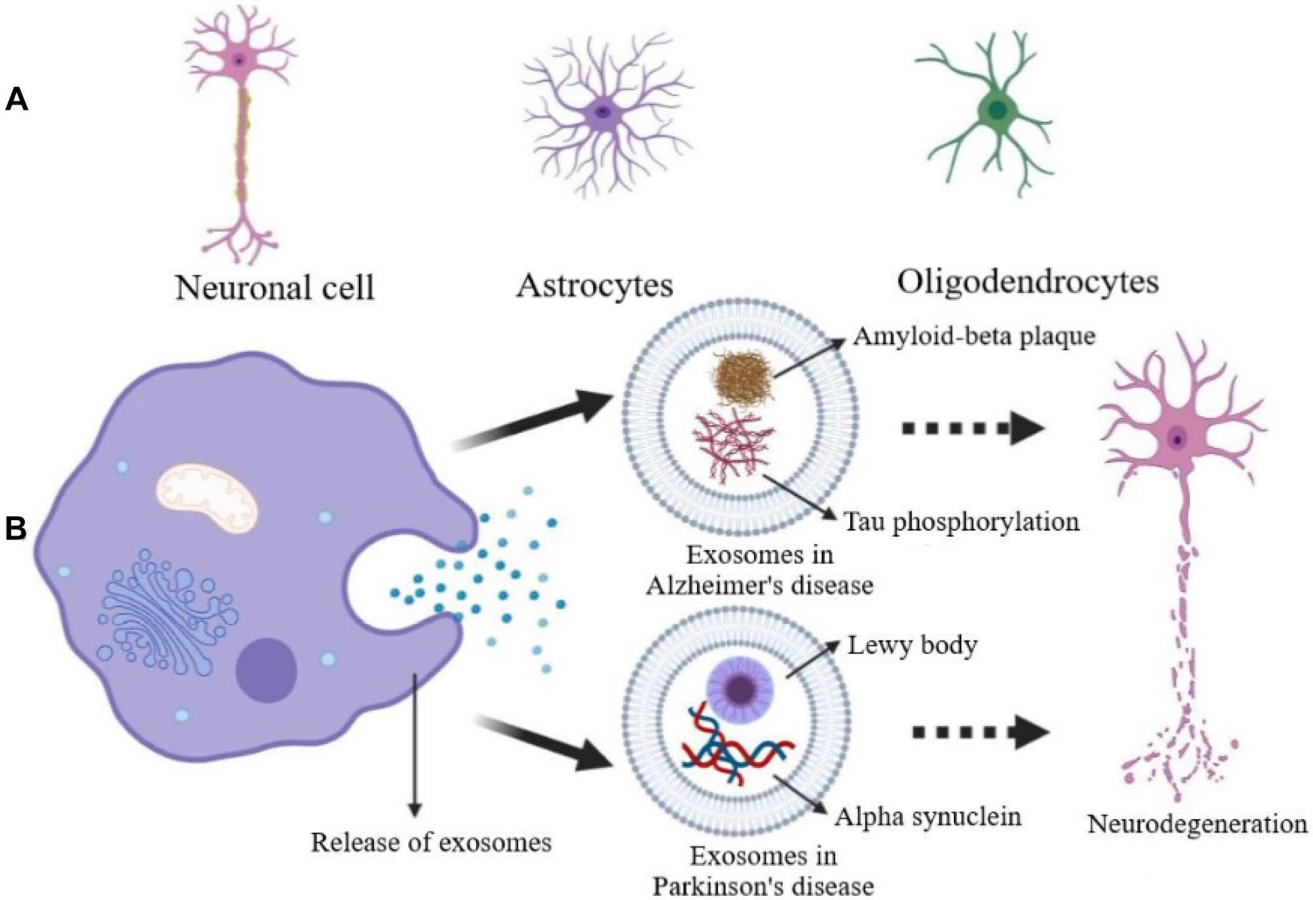
**A** Origin of exosome in neurodegenerative diseases. **B** Release of exosomes from various neuronal cells enables diseases-carrying exosome to neighbouring cells, thereby contributing to the further aggravation of diseases

Neurodegenerative disorders are currently one of the prime reasons for dysfunction and in worst cases death and continue to pose a major challenge for its present-day medical management. However, with the emergence of newer modes of drug delivery, exosomes offer great potential not only as a therapeutic method, but also as an invaluable prognostic biomarker for the treatment of various brain pathologies including neurodegenerative disorders [130]. The differential diagnosis of neurodegenerative diseases, such as Parkinson’s, presents a major challenge, particularly during the early stages of the disease. Researchers aimed to develop a profiling approach for the CSF via miRNAs present in exosomes which were procured from patients with Alzheimer’s and Parkinson’s disease. In comparison to healthy controls, there was found to be a significant (*p* < 0.05) upregulation and under regulation in sixteen and eleven exosomal miRNAs, respectively, in the CSF. Considerable overexpression was observed in miR-153, miR-409-3p, miR-10a-5p and let-7 g-3p in the Parkinson’s CSF exosomes, whereas miR-1 and miR-19b-3p displayed a significant reduction in the independent samples. Bioinformatic analysis conducted by DIANA-mirPath revealed that the significant pathways that possessed great quantities of the patterns of miRNA were neurotrophin signaling, mechanistic target of rapamycin (mTOR signalling), ubiquitin mediated proteolysis, dopaminergic synapse and glutamatergic synapse. Messenger RNA transcripts such as amyloid precursor protein, α-synuclein, Tau, neurofilament and RP11-462G22.1 and prostate cancer antigen 3 (PCA3) which are long-coding RNAs were found to be expressed dissimilarly in the CSF exosomes of Parkinson’s as well as Alzheimer subjects. The results obtained from the study revealed that the exosomes with the encapsulated RNA molecules isolated from the CSF were valuable biomarkers with remarkable robustness with respect to the sensitivity and specificity to differentiate Parkinson’s disease from healthy and disease controls [131]. Figure 4 A represents the origin of exosomes in neurodegenerative diseases. Figure 4 B depicts that the release of exosomes from various neuronal cells enables the disease-carrying exosomes to neighbouring cells, thereby contributing to further aggravation of the diseases.

It was also attempted to determine the expression of miRNAs in exosomes present in the serum and to measure the expression of circulating miRNA in Parkinson’s patients. In the current study, the serum of 109 candidates with Parkinson’s disease as well as 40 healthy volunteers was collected and the expression of 24 candidate’s human miRNAs which are clinical biomarkers of Parkinson’s disease was investigated. The exosomes in the serum containing encapsulated RNAs were extracted and subjected to reverse transcription following which the miRNAs present in the serum were quantitatively analysed using the reverse transcription polymerase chain reaction (qRT-PCR), and the analysis of the characteristic curves of the operating receiver was carried out in order to determine the ability of the miRNAs to appropriately distinguish Parkinson’s. Furthermore, validation of the down regulation of miR-19b as well as upregulation of miR-195 and miR-24 in candidates with Parkinson’s were conducted. In comparison to the control subjects, the values for area under the curve for miR-19b, miR-24 and miR-195 were found to be 0.753, 0.908 and 0.697, respectively, thereby indicating that the level of expression of miR-19b, miR-24 and miR-195 in the serum could be extremely beneficial in diagnosing Parkinson’s disease [132]. A study conducted by provided a new perspective for the treatment of Parkinson’s disease by utilizing the exosomes isolated from the human umbilical cord MSC after exposing it to 6-hydroxydopamine in SY5Y cells as well as Sprague–Dawley rats, thereby suggesting the prominent role of autophagy. The induction of autophagy was initiated upon treatment with exosomes which caused the dopamine stimulated SH-SY5Y cells to multiply and also cause inhibition of apoptosis. Moreover, after crossing the BBB in vivo and upon reaching the substantia nigra, the exosomes were found to relieve apomorphine-induced asymmetric rotation, decrease the depletion of the dopaminergic neurons as well as result in the upregulation of dopamine present in the striatum. Therefore, the findings of this study indicated the prospects of these stem cell–derived exosomes for the efficacious therapy of Parkinson’s disease [133].

In an attempt to identify the pathogenic role of long non-coding RNAs (lncRNAs) in the development of Parkinson’s, the dissimilarities in the expression of these lncRNAs in the peripheral blood exosomes of patients with Parkinson’s were studied. The level of lncRNAs isolated from the plasma exosomes was determined by next-generation sequencing along with real time PCR. The results revealed the upregulation and downregulation of 15 and 24 exosomal lncRNAs, respectively, in the patients with Parkinson’s and also indicated the involvement of lnc-MKRN2-42:1 in the development and progression of Parkinson’s [134].

Due to the absence of an appropriate blood test to distinguish Parkinson’s disease from atypical Parkinsonian syndrome, researchers assessed the practicality of serum neuronal exosomes as a biomarker. The results of the study indicated a twofold increase in the α-synuclein present in the neuronal exosomes in Parkinson’s disease patients in comparison to other neurodegenerative pathologies. The exosomal α-synuclein demonstrated an excellent capacity in the differentiation of Parkinson’s disease across various populations. In patients with non α-synuclein proteinopathies, there was also found to be an elevation in the clusterin exosomes. Thus, the findings of the study indicated the effectiveness of determining the presence of α-synuclein and clusterin exosomes as an exceptional method for the differentiation of Parkinson’s disease from atypical parkinsonism for at-risk populations [135]. Another, study aimed to identify certain serum exosomes as biomarkers of various stages of Parkinson’s by analysing the exosomal proteins using mass spectrometry. The proteonomic analysis revealed a dwindled level for certain proteins such as complement C1q and protein immunoglobulin lambda variable 1–33 (IGLV1-33) cluster-33 in Parkinson’s which could be an indicator to the pathophysiological mechanism of Parkinson’s (137). Other group of research aimed to illustrate the significance of fibroblast growth factor-2 (FGF2) stimulated increase of Ras-associated binding (Rab) proteins in the exosomes in Parkinson’s disease. From a total of 235 relevant proteins that were upregulated, the aforementioned growth factor particularly enhanced the levels of Rab8b and Rab31 in the extracellular vesicles. Furthermore, an investigation was carried out to determine the influence of these Rab proteins in the enrichment of proteins connected with Parkinson’s disease in the CNS, various parts of the brain and in the enteric nervous system through a protein–protein interaction network. A majority of the interactions were obtained for the two Rab proteins, thus demonstrating their relevance in the regulation of the exosomal proteins and its capability to manage the pathophysiology of Parkinson’s disease [137].

In order to develop a more biocompatible method for the treatment of Parkinson’s, researchers utilized blood exosomes for the delivery of drugs across the BBB. The exosomes displayed a nanosize and were deftly loaded into the blood exosomes through a saturated solution incubation technique. The distribution of dopamine in the brain as well as the therapeutic efficacy of the blood exosomes were greatly enhanced in the mouse model of Parkinson’s, thereby indicating the effectiveness of the exosomes for the treatment of Parkinson’s disease [35]. The interaction of two presynaptic proteins, neuronal pentraxin 2 (NPTX2) and neurexin 2a (NRXN2a), along with their respective postsynaptic functional partners, GluA4-containing glutamate (AMPA4) receptor and neuroligin 1 (NLGN1), results in the enhancement of excitatory synaptic responses in certain hippocampal and cerebral cortex regions. Since impairment of the abovementioned excitatory synapses results in the loss of cognitive functions during the onset of Alzheimer’s, the quantification of the neuronal plasma exosomes of the aforementioned synaptic proteins was carried out in order to determine their biomarker properties. There was found to be a significant reduction in the exosomal contents of all 4 proteins in patients suffering from dementia due to Alzheimer’s in addition to cognitive loss which could be attributed to the reduced levels of AMPA4 and NLGN1. Therefore, diminished quantities of such excitatory synaptic proteins could reflect the magnitude of cognitive deprivation and could also possibly indicate the seriousness of the ailment [138].

The researchers also investigated the small RNA contents of the exosomes derived from the brain and their effective use for the determination of pathological changes in the onset of Alzheimer’s disease and its use as an early diagnostic blood test. The exosomes which were derived from the frontal cortex of the brain of Alzheimer’s patients were found to contain an elevated level of miRNA, which could provide a pattern of the biological pathways affected in the course of the disease [139]. It was presented in another study, the possibility of ameliorating the cognitive function in mice induced with Alzheimer’s through the delivery of curcumin-primed exosomes which had enhanced solubility, stability and bioavailability. Due to the interactivity between the exosomes inherited LFA-1 and endothelial ICAM-1, the exosomes were able to easily penetrate the BBB, thereby distributing a huge amount of curcumin in the hippocampal region. The curcumin exosomes were also able to inhibit the tau phosphorylation taking place by the serine threonine–specific protein kinase (AKT/gsk-3β) pathway which resulted in the enhancement of memory and learning disabilities in the mice with Alzheimer’s, thereby acting as a potential method in the therapy of Alzheimer’s [140].

A group of researchers isolated exosomes from the astrocytes of Alzheimer’s disease patients through immunochemical methods and were further matched with controls for the quantification of complement proteins by enzyme-linked immunosorbent assay to determine the mechanism of astrocyte inflammation. Astrocytes are abundantly present in glial cells of the CNS that possess a very important neuronal trophic function through a variety of homeostatic mechanisms. Many neurodegenerative, inflammatory as well as ischemic conditions of the nervous system are known to produce well synchronized multicellular reactions which cause a rise in the number of astrocyte cells, thereby affecting their differentiation into either inflammatory type or ischemia-related type. From the results obtained, it was deduced that the production of antibody-dependent enhancement (ADE) complement effector proteins could be attributed to dysregulated systems in patients with Alzheimer’s disease and were found to be elevated as compared to the controls and also had the potential to cause neuron damage in the late inflammatory stages of Alzheimer’s disease. The current work proposed the pathogenic role of A1 type astrocytes in Alzheimer’s disease due to the presence of inflammatory complement proteins which attained high levels in Alzheimer’s patients in comparison to the matched controls. The reduced amount of various complement regulatory proteins in the early phases of Alzheimer’s, points towards the inhibitory loss of the classical and alternative complement pathways and could be a major reason for complement-mediated neuroinflammation in Alzheimer’s disease. The data obtained also indicated that the current complement-directed therapies could be beneficial to the patients with Alzheimer’s, having elevated levels of complement-mediated neuroinflammation [141].

The researchers suggested a novel approach to pack miR-29 in exosomes, and to further administer the modified vesicles to avoid certain memory deficiencies in Aβ-treated model rats. In order to prevent the flare up of an immune response, the exosomes were derived from the stromal cells of rat bone marrow. The results indicated an increase in the amount of miR-29b, thereby resulting in the downregulation of its target genes beta-site amyloid precursor protein cleaving enzyme 1 (BACE1) and BIM M (Bcl-2 interacting mediator of cell death (BCL2-like 11)), after treatment of the U87 cells with miR-29b-based exosomes. Based on the data obtained, injection of the exosomes, containing miR-29b, conferred protective properties against amyloid pathogenesis, thereby proving the successful outcome of this miRNA-based therapy in the pathogenesis of Alzheimer’s disease [142].

The avenues for the treatment of Alzheimer’s disease were further enhanced by the study which attempted to investigate the regulatory role of exosomes which were isolated from human umbilical cord MSC developed in a 3D culture. The supernatants from these 3D cultures were procured to isolate the exosomes. From the results obtained from the study, it was deduced that the 3D cultured exosomes upregulated the expression of a secretase and downregulated that of b-secretase, thereby reducing the production of Aβ in the pathogenic Alzheimer’s cells as well as in transgenic mice, thereby enhancing the therapeutic response on the improvement of memory and cognitive deficiencies in mice with Alzheimer’s [143].

Exosomes derived from the MSC were also used to enhance the neurogenesis as well as the cognitive capabilities in a mouse model for Alzheimer’s disease. The results of the study indicated that the exosomes isolated from the stem cells were able to augment neurogenesis in addition to restoring the cognitive function which was decreased on administration of Aβ1-42 aggregates [144]. Similarly, the study illustrated the benefits of exosomes derived from the human umbilical cord MSC which were found to alleviate the cognitive dysfunction and eliminate the Aβ deposits in the mice. The exosomes also had inflammation regulatory effects to activate the microglia and regulate the inflammatory cytokine levels in in vitro [145].

Another novel method was developed which utilized the concentration of salivary exosomes as a correlation measure for the degree of cognitive impairment using nanoparticle tracking analysis technique in Alzheimer’s patients. On measurement of the total exosomes present in the saliva, the results indicated significant differences in the concentration of the exosomes between patients suffering from Alzheimer’s and the healthy controls. A similar result was also observed upon validation using an exosome surface marker, i.e. CD63. The findings were further confirmed by correlation with the expression levels of oligomeric amyloid-beta and phosphorylated-tau protein obtained from salivary exosomes. It was also observed that the phospho-tau, Aβ oligomer/fibril and Aβ protein was abundantly present in patients with Alzheimer’s and the cognitive impairment as opposed to their healthy counterparts. Therefore, the results of the study indicated that the quantity of salivary exosomes, obtained through nanotracking method, presents prospective use as a cost-effective method in the early diagnosis of Alzheimer’s [59].

A novel technique for the longitudinal and quantitative in vivo neuroimaging of exosomes was recently developed by group of researchers using gold nanoparticles as a labelling agent in combination with the superior visualizing capability of the classical X-ray computed tomography. This method was utilized to track the patterns of homing and migration after the intranasal delivery of the MSC-derived exosomes of the bone marrow in various CNS diseases. It was observed that the exosomes were specifically targeted in the pathological areas of murine model brain regions and were accumulated up to a time period of 96 h post-administration, whereas the healthy counterparts exhibited a more diffused migratory route and clearance within 24 h. There was an extremely high correlation between the neuroinflammatory signal in pathological brains and exosomal accumulation, thereby proposing the mechanism to be inflammatory driven. Moreover, the MSC-derived exosomes exhibited selective uptake by the neuronal cells in the pathological sites but not glial cells. All in all, the results of this study contribute significantly to the use of exosomes in the diagnosis and therapy of numerous CNS disorders including Alzheimer’s and Parkinson’s diseases [146].

The study also involves proteomic analysis of the exosomes derived from the CSF of patients suffering from amyotrophic lateral sclerosis in order to identify new biomarkers associated with the disease. Liquid chromatography-tandem mass spectrometry of the fluid fractions of amyotrophic lateral sclerosis (ALS) subjects was conducted utilizing gel filtration chromatography. Novel INHAT repressor (NIR) protein was found to be greatly elevated in ALS patients whose dysfunction could possibly contribute to the nucleolar stress in sporadic ALS pathogenesis [147].

Researchers investigated therapeutic effect of exosomes derived from adipose stem cells in the SOD1(G93A) murine model of mice. The effectiveness of the exosomes through the intravenous and intranasal route was also determined. The findings of the study revealed an improvement in the motor capacity as well as protection of the lumbar motoneurons and muscle cells. There was significant reduction in the activation of glial cells in the murine models thereby proving it as a potential treatment for amyotrophic lateral sclerosis in humans [148].

In order to determine the extent of inflammation in patients with ALS, a study conducted involves the concentration of interleukin-6 (IL-6) in exosomes that were derived from astrocytes. An increase in the levels of IL-6 in the exosomes was indicative to sporadic ALS but was limited to subjects having the disease for less than a year [149]. It was aimed to identify biomarkers for ALS through a sensitive next-generation sequencing method. Around 543 genes were found to be changed in the exosomal mRNAs in patients with ALS, represented by the gene CUEDC2, thus proving to be a potential biomarker in ALS [150].

Another genetic neurodegenerative disease that has been treated through the use of exosomes is Huntington’s disease (HD). HD is caused due to an abnormality in the expansion of cytosine, adenine and guanine (CAG) repeats that is responsible for encoding the gene huntingtin. This results in a myriad of neurological symptoms including the cognitive impairment, involuntary choreiform movements and neuropsychiatric conditions. Though the exact mechanism of action of neurodegeneration in Huntington’s disease remains unclear, it could be attributed to alterations in RE1-silencing transcription factor (REST) which is a transcription regulator. This results in inability of the mutant huntingtin to silence the activity of REST, thereby causing enhanced binding of REST to a neuron restrictive silencer element which results in transcriptional dysfunction. Other researchers used exosomes as a means of delivery in the treatment of this degenerative condition. One of the prime miRNAs, i.e. miR-124, which is repressed in Huntington’s disease was observed to be overexpressed in a stable cell line from which the exosomes were further harvested. The exosomes were found to be greatly express miR-124 expression, which later were taken up by the recipient cells. After injecting the exosomes into the striatum of R6/2 transgenic HD mice, the expression of the target gene, RE1-silencing transcription factor, was found to decline. Although, the miR-124 based exosomes did not result in significant behaviour improvement. The current investigation served as conceptual evidence for the delivery of miRNA using the exosomal method of administration for neurodegenerative diseases [151]. Table 2 depicts exosomes used as potential diagnostic markers for various brain neurodegenerative disorders.

**Table 2:** Exosomes used as potential diagnostic markers for various brain neurodegenerative disorders

Disease	Objective	Activity	Outcome	References
Parkinson’s disease (PD) and Alzheimer’s disease (AD)	MicroRNA profiling method for exosomal miRNAs in the cerebrospinal fluid (CSF)	miRNA profiles of exosomes present in the cerebrospinal fluid of PD and AD patients	Elevated levels of miR-153, miR-409-3p, miR-10a-5p and let-7 g-3p in the CSF	[131]
PD	Investigation of 24 miRNAs as diagnostic tools	Quantitation of serum miRNAs by qRT-PCR	miR-19b, miR-24 and miR-195 acted as biomarkers for Parkinson’s	[132]
AD	Determining the effect of AD on the levels of plasma neuronal exosomes	Quantification of plasma neuron derived exosomes for biomarker properties	Diminished levels of the proteins in dementia due to Alzheimer’s	[121]
AD	Investigation of astrocyte inflammatory mechanisms	Enzyme linked immunosorbent assay quantification of the complement proteins of exosomes from AD patients and controls	Levels of astrocyte-derived exosomes were higher at the dementia stage, and complementary proteins CD59 and CD46 were diminished	[141]
Huntington’s disease (HD)	Delivery of abnormal downregulated miRNAs to normalize gene regulation	Potential of exosome-based delivery of miRNA in HD was analysed	Injection of Exo-124 in mice reduced expression of RE1-silencing transcription factor	[151]
PD and AD	Development of in vivo neuroimaging of exosomes using gold nanoparticles	Tracking of the migration patterns of MSC-derived exosomes	MSC-derived exosomes targeted the pathologically relevant sites in murine models upto 96 h	[146]
AD	Correlation of salivary exosomes with progression of AD	Concentration of salivary exosomes in Alzheimer patients were compared to that of healthy controls	Nanoparticle tracking analysis method revealed differences in salivary exosomes in Alzheimer patients and healthy controls	[59]
PD	Determination of capability of human of umbilical cord MSCs in the treatment of PD	Determination of the ability of human umbilical cord MSC-derived exosomes to offer neuroprotection	Exosomes reduced the loss of dopaminergic neurons in the substantia niagra along with upregulation of dopamine	[133]
PD	Investigation of the effect of long non-coding RNA in blood exosomes in the pathogenesis of Parkinson’s	Evaluation of the levels of lncRNAs in exosomes of PD patients and healthy controls	Long non-coding MKRN2-42:1 revealed positive correlation with UPDRS III score in Parkinson’s subjects	[134]
PD	Assessment of serum neuronal exosomes as biomarkers	Levels of neuron-derived exosomal α-synuclein and clusterin was analysed	Neuron-derived exosomal α-synuclein and clusterin was found to be elevated in PD with AUC = 0.98	[135]
AD	Determination of therapeutic effect of exosomes containing miR-29b in rat model	Investigation of the expression levels of miR-29b in a rat model	Transfected cells displayed overexpression of miR-29 along with downregulation of target genes	[142]
PD	Demonstration of the effect of fibroblast growth factor-2 (FGF2) in the release of exosomes enriched with Rab proteins	Expression levels of Rab8b and Rab31 enriched exosomes due to FGF2 were analysed	Significant upregulation in the levels of exosomes with Rab8b and Rab31 due to FGF2	[137]
AD	Determination of the therapeutic effect of exosomes derived from 3D culture of stem cells	Evaluating the level of regulation of exosomes derived from a 3D culture	Exosomes derived from the 3D culture altered the expression of α-secretase and β-secretase	[143]
AD	Fingerprinting of small RNA content in extracellular vesicles	Analysis of small RNA content in exosomes derived from the frontal cortex	Upregulation of disease-associated miRNA in exosomes derived from the brain	[139]
PD	Proteomic analysis of serum exosomes and identification of biomarkers	Analysis of the protein’s levels of serum exosomes	Reduced expression levels of proteins such as apolipoprotein D and J and afamin in PD patients	[136]
AD	Examining the effect of MSC-derived exosomes in AD mouse model	Effect on recovery of cognitive function in AD mouse model	MSC-derived exosomes alleviated cognitive impairment due to beta amyloid 1–42	[144]
AD	Design of curcumin exosomes to alleviate symptoms of AD	Potential of curcumin exosomes to prevent hyperphosphorylation in AD mice was analysed	Curcumin exosomes caused inhibition of Tau phosphorylation	[140]
Amyotrophic lateral sclerosis (ALS)	Proteomic analysis of CSF exosomes of ALS patients	Identification of proteins that were linked to ALS was conducted	Novel INHAT repressor (NIR) protein levels were diminished in the motor neurons of patients with ALS	[147]
ALS	Amelioration of ALS progression using adipose stem cell–derived exosomes	Determination of the neuroprotective effects of adipose stem cell–derived exosomes	Improvement of motor performance and reduced activation of glial cells	[148]
ALS	Determination of biomarker characteristics of IL-6 in ALS	Analysis of expression levels of IL-6 in ALS patients	Increased levels of IL-6 in astrocyte-derived exosomes of ALS patients	[149]
ALS	Biomarker identification in CSF of ALS patients	Comprehensive analysis of exosomal RNAs in the CSF of ALS patients	CUEDC2 was suggested to a biomarker candidate for ALS	[150]
AD	Examining the effect of exosomes to reduce neuroinflammation in AD	Analysis of the potential of exosomes to diminish amyloid beta deposits and reduce neuroinflammation	Stem cell–derived exosomes were able to eliminate the deposition of Aβ in mice	[145]
PD	Development of a biocompatible method for delivery across the blood–brain barrier	Potential of blood exosomes loaded with dopamine for Parkinson’s therapy	Distribution was enhanced by 15-fold in the brain	[35]

### Exosomes in the diagnosis and treatment of brain tumour

Exosomes are rich in cargos as they contain higher amounts of nucleic acids and proteins, which directly reflect the metabolic state of the cells. Differential expression and disrupted homeostatic features of exosomes help in cargo trafficking against several diseases like cancer. Increased release of exosomes leads to oncogenic progression and metastases. Exo-miRNA can communicate with the adjacent cells of the same tissue or neighbouring tissue through gap junctions. This is termed as the “bystander effect”. This effect leads to autophagy of the cells and makes the cells more cancerous [152, 153]. Recent studies have shown that exosomal RNAs interfere even in cell migration, proliferation, metastasis, angiogenesis, apoptosis and chemo-resistance [154–158].

#### Role of exosomes in glioblastoma

Glioblastoma (GBM) is a malignant tumour with several cells protruding deep into the cerebral lobes of the brain. Exosomes play a very prominent part in glioblastoma. They remain enclosed with oncogenic proteins which cause the spreading of the tumour to the neighbouring tissues. These tumours also show miRNA upregulation [42]. Exosomes help in the transfer of oncogenic activity, the transformation of phenotype and epidermal growth factor receptor (EGFRvIII)–dependent transcription by transporting the oncogenic receptor, EGFRvIII amid glioma cells [159]. Exosomes linked with DNA fragments like sequences of N-ras oncogenes and full-length H-ras were reported as the exosomal contents, released by mouse brain tumour cells [160]. Glioblastoma cells also secrete MVs enclosing miRNA, angiogenic proteins and mRNA [42]. The target endothelial cells form tubule when the host human brain microvascular endothelial cells engulf the mRNA molecules. Both the tumour and its associated cells secrete exosomes which favours the tumour growth by transferring pro-tumourigenic factors. For example, glioma cells with EGFRvIII can promote the recipient cell growth by activating the mitogen-activated protein kinase (MAPK) and Akt signalling pathways [159].

#### Exosomes as diagnostic tools for brain tumour

In the last few years, it has been discovered that milk, blood, urine and saliva contain exosomes, and as they have higher amounts of RNA, lipid and specific protein content, they became useful in the diagnosis of several diseases. Blood borne miRNAs are very imported biomarkers as reported by some groups in 2008. As naked RNA was found degrading faster and faster in blood, it was supposed that these RNAs must be protected by few macromolecular complexes which later came to be the “exosomes” [161]. Nanoscale flow cytometry, which was conducted pre-density gradient ultracentrifugation (DGU), demonstrated that glioma patients showed increased MVs along with efficient exosome isolation stating them as potential biomarkers for the diagnosis of tumours. This resulted in global immunosuppression rather than an increase of circulating tumour-immunosuppressive exosomes [162]. A new technique has now gained importance in recent years, which favours the overtime surveillance of brain tumours by identifying the GBM-specific exosomes in CSF or blood. This gives the exact characterization of the tumour. This technique is called as “liquid biopsy” [163]. It was identified that a fundamental biomarker named syndecan-1 can distinguish between low and high-grade tumours in glioblastoma [164]. Besides, excessive levels of miRNA are related to tumour progression, while high miR-21 expression suggests increased invasive capacity [165].

CSF contains large amounts of tumoural exosomes, because they need not cross the BBB to enter the CSF and so it is less contaminated with platelet-derived exosomes. So, a more convenient means of sample collection is blood. Therefore, the most specific sample for diagnosis of GBM is still in difficulty [166]. It was demonstrated that exosomes with the H63D human homeostatic iron regulator (HFE) variant of the HFE gene can promote a more angiogenic situation in WT HFE cells along with increased cancer cells [167]. A lower-grade glioma can be diagnosed by the presence of 23 dysregulated miRNAs in mutations in isocitrate dehydrogenase 1 and 2 (IDH^MUT)^ samples. It was reported that free-circulating miRNAs of GBM patients help in diagnostic purposes rather than exosomal miRNAs [168]. It was demonstrated that exosomes with miR301a, separated from grade IV glioma patients, showed an increase in invasion and proliferation of H4 glioma cells [169]. Different exosomal cargos from GBM patients which act as potential diagnostic markers for glioblastoma are listed in Table 3.

**Table 3:** Exosomal cargos as potential diagnostic markers and therapeutics for glioblastoma

Disease	Objective	Activity	Outcome	References
Glioblastoma multiforme (GBM)	Investigation of safety and efficacy of monoclonal antibodies targeting the PD-1/PD-L1 axis	Preclinical studies on PD-1 expression in GBM mouse models	Significant tumour regression and long-time survival of animals	[208]
Proneural GBM	Effect of IDH-1 mutations, 1p19q deletion, MGMT promoter methylation and EGFRvIII amplification in patients	Frequent testing of IDH-1 mutants in routine clinical practice in GBM patients	Retardation of DNA repair in tumour cells by inducing apoptosis	[209]
Glioblastoma	Investigation of the role of GSC-EXs in promoting the angiogenic function of ECs through the miR-21/VEGF signal	Quantification of total mRNA by RT-PCR and VEGF signals by ELISA	Triggered the angiogenesis by stimulating the VEGF pathway	[210]
GBM	Detection of the enhancement of chemosensitivity to temozolomide through exosomal transfer of miR-151a	Direct targets of miR-151a were identified by microarray assays, bioinformatics and further RNA chromatin immunoprecipitation (RNA-ChIP) assay	In formerly normal cells, temozolomide resistance was induced	[211]
GBM	Diagnosis of chemoresistance in GSCs	Induction of CD133, CD44	Acted as probable markers for chemoresistance	[212]
GBM	Investigation of the effect of the hypoxic microenvironment and adenosine on MDR mechanisms	Induction of MDR mechanisms	Induced chemoresistance phenotype	[212]
GBM	Identification of markers like ANXA1, ITGB1, CALR, PDCD6IP, PSMD2, ACTR3, APP and CTSD in aggressive tumours by using glioblastoma derived extracellular vesicles	EVs secreted by GBM cells were isolated and analysed by quantitative high-resolution mass spectrometry	Stimulated invadopodia and favoured invasive capacity	[213]

#### Exosome-based glioblastoma therapy

Many phase I studies were conducted in the 2000s for exosomes. The first study was the vaccination of metastatic melanoma patients with autologous dendritic cell (DC)–derived exosomes (DEX). These were generated by adding functional major histocompatibility complexes (MHC) capable of promoting T cell immune responses along with tumour rejection [170]. The present use of exosomes for the treatment of brain tumours is mainly classified into three major categories. They are exosome-based immunomodulation therapy [152], exosomes as delivery vehicles for anti-tumour nucleotides [153] and exosomes as drug delivery vehicles [153, 171]. Several researchers also focussed on the treatment of brain tumours using exosomes as other treatments are showing a few side effects. The exosome number increases by radiotherapy and poses a serious threat to the surrounding cells of the tumour. Thus, radiotherapy serves as a pre-treatment for the uptake of therapeutics-enriched exosomes [172]. It was demonstrated that intranasal administration of catalase-loaded exosomes resulted in the behavioural recovery of a murine model with Parkinson’s disease showing that exosomes can cross the BBB for the therapy of brain tumours [173]. Recent studies have demonstrated that exosomes isolated from host cells transfected with miRNA-encoding vectors can deliver the target miRNAs in animal models [174]. For example, miR-146B-overexpressing and miR-122-overexpressing exosomes from engineered MSC inhibited glioma growth in rat brain [175]. It was reported that the delivery of short interfering RNA (siRNA) to mice brain was achieved by the aid of exosomes, which was targeted through engineered DC expressing Lamp2b (a membrane protein present in exosomes) fused with neuron-specific rabies virus glycoprotein (RVG) peptide 3 [34]. It was found that exosomes incorporated with paclitaxel had significantly increased the cytotoxicity of brain tumour cell lines, U-87, while the empty exosomes showed cell viability [176]. Exosomes derived from natural killer (NK) cells had targeted and resulted in anti-tumour effects in both in vitro and in vivo, stating their utility in treating inoperable glioblastoma. It was proved that cRGD-loaded paclitaxel exosomes had significantly improved the curative effects of paclitaxel in glioblastoma multiforme through enhanced targeting [177]. At present, a few exosome-based therapeutics are still in phase II clinical trials not only for the treatment of brain tumours, but also for different types of cancer.

### Exosomes in the diagnosis and treatment of neuroinflammatory diseases

Neuroinflammation is an innate immune response induced by microglia and astroglia when they are triggered by various damage stimuli. Neuroinflammation results in the production of reactive oxygen species, chemokines, secondary messengers and cytokines [178]. Neuroinflammation is a prevalent feature of numerous neurodegenerative diseases like Alzheimer’s disease, amyotrophic lateral sclerosis and Parkinson’s disease. All these diseases are certainly characterized by a high level of pro-inflammatory cytokine production and glial activation in the CNS [179]. In the recent era, neurodegenerative diseases became the biggest hardship in the health care system as they are the main cause of disability and death [180]. In the research of neurodegenerative diseases, the major keystone is “intercellular communication”. Apart from the several molecular mechanisms that happen within a cell, the effects that these cells produce on the surrounding cells have become a topic of investigation. Recent studies proved that exosomes are involved in the pathogenesis, diagnosis and therapy of neuroinflammatory disorders.

#### Role of exosomes in neuroinflammatory diseases

**Fig. 5:**
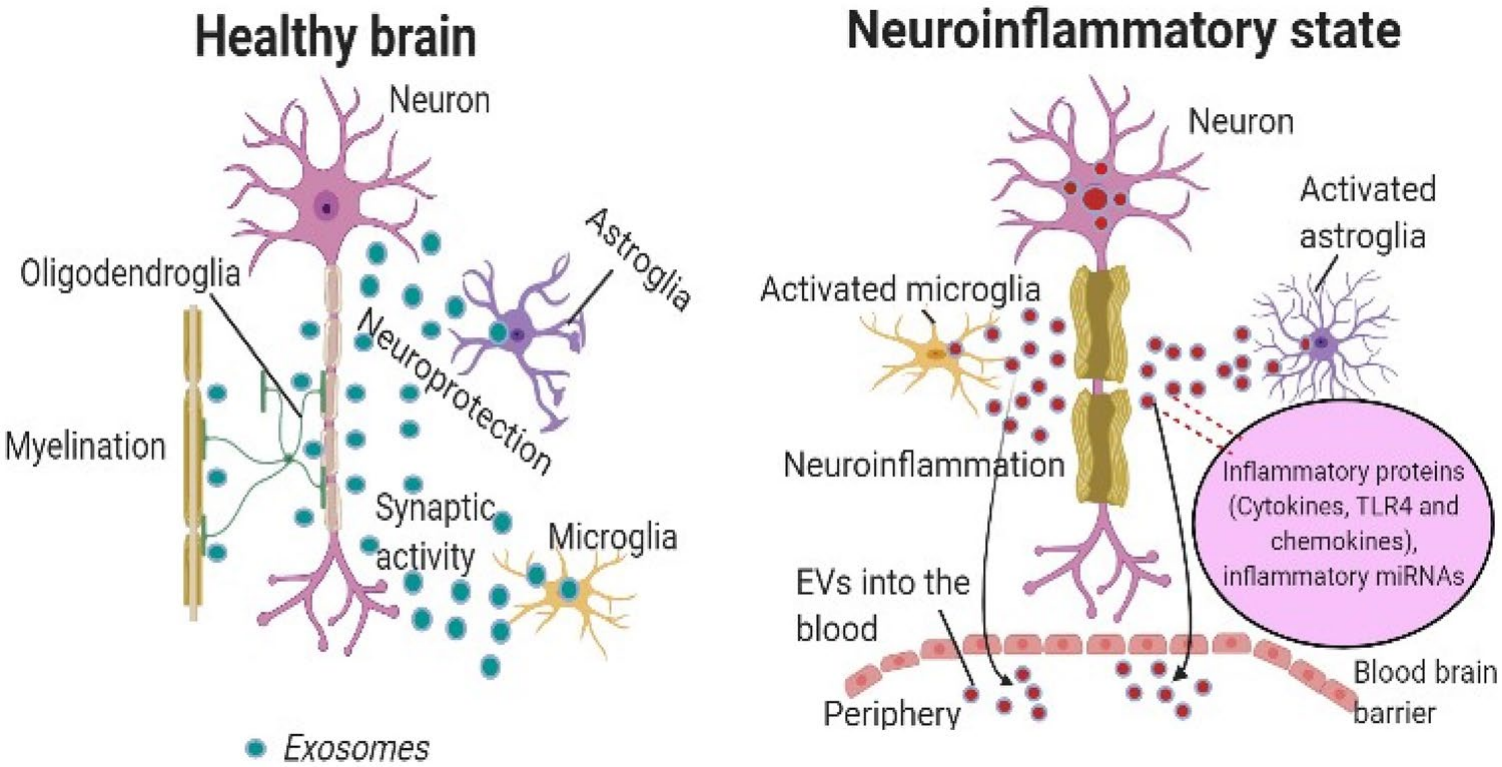
**A** Role of exosome in healthy brain. **B** Release of exosomes in neuroinflammatory state

Exosomes play a very pivotal role in triggering the inflammatory cascade. This is mainly favoured by proteins such as amyloid β, α-synuclein and prions, which move from one cell to another cell [181]. Cross-talk between the central and peripheral nervous systems is due to the stimulation of peripheral immune response associated with inflammation in the central nervous system. Experimental evidence reported that purified serum-derived exosomes from lipopolysaccharide (LPS)-treated mice can induce both CNS and systemic inflammation [179]. Furthermore, after a traumatic brain injury, the excessive inflammatory response increases the neurologic outcome. It was recently established that after brain injury, the miR-124-3p level increases, and this decreased the neuronal inflammation and promoted the neurite outgrowth after scratch injury [182]. Astrocyte-derived exosomes can also transport the misfolded pathogenic proteins and aberrantly expressed miRNAs into neurons which then initiate neuroinflammation resulting in neurodegeneration and death of neurons [98]. Extracellular vesicles can readily cross the BBB, by making a communication channel with CNS through modulating the physiological processes using systemic inflammation. In a healthy brain, glial-derived exosomes mediate main functions participating in neural circuit development and maintenance, promoting neurite outgrowth, neuronal survival and synaptic activity. Atrophic uphold is given by oligodendrocytes-derived exosomes to axons, promoting myelination (Fig. 5A) and role of exosomes in the neuroinflammatory state (Fig. 5B). After a neural mock, microglial and astroglial cells are activated and release exosomes that consist of inflammatory proteins, miRNAs and misfolded proteins involved in a neuroinflammatory response affecting the viability of the neuron. These exosomes can cross the BBB propagating the neuroinflammatory response to the periphery and can be used as potential biomarkers for the pathogenesis of neuroinflammation and neurodegenerative disorders.

### Exosomes as diagnostic tools for neuroinflammatory diseases

Numerous pieces of evidence suggested the potential role of exosomes as vectors containing miRNA, mRNA, lncRNA, peptides, siRNA and synthetic drugs for cell therapy against neurological, neurodegenerative and neuroinflammatory diseases and the biomarkers for brain diseases. Recent studies have demonstrated that patients diagnosed with bipolar disorders or schizophrenia showed differentially expressed miRNAs when compared to the matched controls [183]. Besides, a tumour-specific EGFR transcript variant was detected in the vesicles isolated from cancer patients and this was considered a good diagnostic tool for neuroinflammatory diseases [42]. It was reported that purified LPS-stimulated exosomes from the blood when infused directly into the cerebral ventricles increased the microgliosis significantly. It was also demonstrated that astrogliosis caused by injecting serum-derived exosomes to mice has improved the systemic pro-inflammatory cytokine production and elevated CNS expression of pro-inflammatory cytokine mRNA [179]. It was also reported that neuronal exosomes derived from plasma served as biomarkers for the diagnosis of Alzheimer’s disease and HIV infection [184]. It was demonstrated that plasma astrocyte-derived exosomes (ADEs) and complement protein levels (CPs), as the components of neurotoxic neuroinflammation, served as the predictive biomarkers for the conversion of mild cognitive impairment to Alzheimer’s disease [185]. Acute neuroinflammation and oxidative stress induced by IL-1β generate the specific subset of miRNAs via exosomes which had also helped in the diagnosis of neuroinflammation associated with neurological injuries and disorders [186]. It was reported that Treg-derived exosomes were deficient in inflecting the proliferation and survival of conventional T cells in multiple sclerosis patients [187]. Acid sphingomyelinase–enriched exosomes were found to produce lipidomics in the CSF of multiple sclerosis patients [188].

Furthermore, hippocampal neurogenesis was impaired in mice by the injection of cultured exosomes consisting of known pathogens into the dentate gyrus [189]. Corticosteroids and cytokines may activate the release of astrocytic exosomes with many miRNAs important for stress response, neurogenesis and cell survival [190]. IL34/CD81 ratio was increased significantly in the patients with major depressive disorder (MDD) when compared to control resulting in increased inflammation [191]. It was demonstrated in a recent study that extracellular vesicle release was increased in rats in which central inflammation was detected systematically through exosomal vesicles (EVs). These EVs act as diagnostic markers for mental disorders [192]. It was reported that neural exosomes extracted from plasma have also been used in a pilot study to scrutinize the protein biomarkers for major depressive disorder patients [191].

#### Exosomes in the treatment of neuroinflammatory diseases

Exosomes possess special features that make them the optimal tools to deliver the molecules therapeutically by crossing BBB. They transfer the brain antigens to the periphery and regulate the peripheral immune system [193]. It was shown that exosomes loaded with curcumin not only increased the stability and bioavailability of the compound in vivo, but also increased the survival significantly in lipopolysaccharide-induced septicemia [71]. Exosomes derived from MSC alleviated the traumatic brain injury effects or focal cerebral ischemia in animal models by increasing the neurovascular remodelling and improving behavioural, neurological and cognitive outcomes during recovery [38]. Hypoxia-preconditioned mesenchymal stromal cell–derived exosomes alleviated cognitive impairment in a mouse model of Alzheimer’s disease by protecting synaptic dysfunction, modulating the microglial and astrocytic activity and reducing the pro-inflammatory factors like TNF-α and IL-1β [84]. Several clinical trials are also going on presently to treat the neuroinflammatory diseases by using exosomes. Table 4 gives the information about different exosomal cargos for the therapy of various neuroinflammatory diseases.

**Table 4:** Exosomes from different origins in the treatment and diagnosis of neuroinflammatory diseases

Disease	Objective	Activity	Outcome	References
Alzheimer’s disease	Investigation of the effects of MSC-derived exosomes on neurogenesis and cognitive capacity in a mouse model of Alzheimer’s disease	Neurogenesis in the subventricular zone was determined by immunofluorescence using doublecortin and PSA-NCAM antibodies	Cognitive function was recovered in mouse models	[144]
Neuroinflammation	Effect of exosomes in inflammation cascade	Examined by RT-PCR	α-synuclein, amyloid-β and prions are moved between cells into exosomes triggering an inflammatory cascade	[181]
Traumatic brain injury	Inhibition of neuronal inflammation through increased miR-124-3p in microglial exosomes following traumatic brain injury	Real-time PCR was done in isolated exosomes	Neuronal inflammation was reduced	[182]
Alzheimer’s disease	Alleviation of liver fibrosis by using human umbilical cord derived MSCs	EMT-associated markers were expression was observed in vitro	Alleviated neuroinflammation and reduced amyloid-beta deposition by modulating microglial activation	[79]
Parkinson’s disease	Delivery of designer exosomes produce by implanted cells intracerebrally	Evaluated by using the Nanoparticle Tracking Analysis (NTA) software	Constricted neuroinflammation and neurotoxicity in in vitro and in vivo C57BL/6 J female mice models	[214]
Autoimmune uveitis	Investigation of release of exosomes by Interleukin-35-Bregs	Monitored EAU progression by fundoscopy, histology, optical coherence tomography and electroretinography	Exosomes suppressed neuroinflammation	[24]
Perinatal brain injury	Anti-inflammatory effects of exosomes derived from hWJ-MSC on microglia mediated neuroinflammation in perinatal brain injury	In vitro stimulation of immortalized BV-2 microglia and primary mixed glial cells with lipopolysaccharide (LPS) in the presence or absence of exosomes	Reduced microglia-mediated neuroinflammation	[215]
Cerebral ischemia	Regulation of neuroinflammation by glutaminase 1	Analysed by immunocytochemistry and Nanoparticle Tracking Analysis (NTA) software	Regulated neuroinflammation by enhancing microglial activation and release of pro-inflammatory exosomes	[216]

### Exosomes in the diagnosis and treatment of brain meningitis, encephalitis and CNS tuberculosis

Protection of the brain was shown by the treatment of the animal (mice) model of oligodendrocyte glycoprotein peptides induced experimental autoimmune encephalitis with the use of exosome-loaded curcumin through the intranasal path. This was observed due to the selective uptake by microglial cells of the curcumin entrapped exosome and thus resulted in apoptosis of the cells [71]. In a research on the immunological function of exosomes in autoimmune encephalitis (AE), exosomes expressing neuronal auto-Ags were found to be present in CSF of patients with antibody-positive AE. It was concluded that exosomes carrying neuronal auto-Ags would play an important role in immune pathogenesis of it [194]. Human guanylate binding protein-1 is a secreted GTPase that is present in patients with bacterial meningitis at elevated CSF concentrations. In combination with exosomes, this protein may be present and may therefore be used as a surrogate biomarker for bacterial meningitis [195]. Exosomes can also help in the treatment of bacterial meningitis [196]. Central nervous system tuberculosis (CNS-TB) is the most life-threatening of all types of TB, causing high mortality and morbidity even with effective anti-TB treatment. In a review of progress in the diagnosis and treatment of CNS tuberculosis, it was concluded that the exosome targeting drug delivery system would play a greater role in the diagnosis and treatment of CNS tuberculosis, taking into account the benefits of exosomes (ability to cross the BBB and high drug loading) [197].

## Routes of administration of exosomes in the diagnosis and treatment of CNS disorder

Human stem cell–derived extracellular vesicles such as exosomes offer numerous benefits in comparison to cell-based treatments for the diagnosis and therapy of functionally impaired tissues and organ sites [198]. Exosomes can be administered through various routes such as the oral, intranasal, intraperitoneal, intravenous, intraventricular, subcutaneous and intertumoural route depending on the concerned disease state. Various methods such as fluorescence labelling, radionuclide and magnetic resonance imaging are used for the imaging and as a tracking mechanism for exosomes. However, the fluorescence dyes used in fluorescence labelling have been found to be deposited in the tissues even after degradation of the exosomes [199]. The intravenous and intranasal routes represent the most popular methods for administration of exosomes to be targeted to the CNS. The intravenous route has been used to administer exosomes to various organs such as the heart, lung, kidney, liver and brain. Owing to their endogenous origin, the intravenously administered exosomes avoid elimination by the immune system and hepatic clearance to the same magnitude as that of exogenous nanoparticles, thereby allowing the recipient cells sufficient time for the uptake of a high tissue dose [199]. The systemic routes present a more feasible approach for the administration of exosomes thereby negating the need for invasive surgical procedures. Recent studies show that systemic administration routes such as the retro-orbital vein and intranasal delivery demonstrate the effective penetration and perfusion of exosomes in the least invasive way throughout the brain, thus pointing to a more translationally tractable method for the treatment of various neurological pathologies [198]. Recently, researchers have also conducted comparative studies to determine the effect of the intravenous and intranasal route in the delivery of adipose-derived stem cells in amyotrophic lateral sclerosis. It was observed that repeated administration enhanced the motor performance; protected the lumbar motor neurons, the neuromuscular junction and muscle; and decreased the glial cells activation in the animals that were treated with exosomes. From the results obtained, it was concluded that the intranasal route exhibited superior properties over the intravenous route, since the labelled exosomes administered by the former route were detected in the brain, thereby indicating the presence of the vesicles in the CNS which were accumulated in the lesions of the brainstem motor nuclei in the murine model of amyotrophic lateral sclerosis [148]. Furthermore, another research group performed a comparative study between three distinct routes of administration, i.e. the intrahippocampal transplantation, retro-orbital vein injection and intranasal route for the delivery of human neural stem cell–derived extracellular vesicles that were isolated from conditioned media. The ability of the exosomes to translocate into normal tissue in the brain was determined after administration [198]. It was observed that, although the net quantity of vesicles was constant, each of the administration routes required different volumes for appropriate biological distribution. From the data obtained, it was deduced that all the aforementioned administration routes were functionally equivalent to deliver the vesicles to the brain parenchyma with no significant differences in the amount of vesicles to translocate across the brain [198].

## Challenges in marketing and commercialization of exosome for brain diseases/disorders

So far, there are no clear regulatory criteria for pharmaceutical production and clinical application, considering the growing interest in these carriers. However, it is unavoidable that their production should be carried out strictly in accordance with GMP (good manufacturing practice), GLP (good laboratory practice), GDP (good documentary practice), GCP (good clinical practice) and GSP (good storage practice) in a specific well-organized structure in order to eliminate any possible contamination and take into consideration both donor and recipient safety [200]. The introduction of prepared formulation to the market and clinics is confronted with various challenges. Exosomes have a tough process from laboratory to market as they are delivery system based on nanotechnology with a significantly high-level risk of commercialization. In their introductory route to the market and the clinics, there are many serious challenges, including technological, economic and regulatory issues. The instability of nanoscale materials, their complex design, production, storage, scale-up process, in vivo studies, clinical trials and variation from batch to batch are some of the factors that cause these promising nanovesicles to be used. Although, professionals should estimate costs, profit-to-risk ratio, order to maximize production, storage conditions and scale-up methods. Regulatory authorities are currently evaluating emerging nanodrug product by product. This approach contributes to the lack of an established norm for practitioners and firms. Also, any additional ethical questions could be in the way as exosomes are sourced from the cells. In personal medicine, nanopharmaceuticals such as exosomes have a great potential to be used, which can be considered in exosome marketing. In order to coordinate research, some guidelines have been prepared by the international society for extracellular vesicles suggesting limited details to be given in extracellular vesicles (EV) studies. In the future, given the successful efforts made to check the exosome potential and bring them to clinical trials, much progress will be anticipated in this field [45].

## Factors affecting clinical use of exosome for brain diseases

In the past decade, many clinical trials have attempted to use exosomes in clinical therapy, but questions about its optimization remain unanswered, such as how to increase the exosome yield specific to the cell type and maximize output which is to be considered in using exosomes for the brain. In addition, how to store broad therapeutic substances in and on exosomes and how to design a better delivery procedure in clinical practice for particular tissues are uncertain. Following are the factors that affect the clinical use of exosomes:

1. Source: Parental cells from where exosomes are sourced are an important consideration in developing therapeutic exosomes. Its critical technologies should be addressed, and associated side effect should be identified [201], for example, MSC-derived exosomes for cancer therapy [202, 203].
2. Biodistribution: The natural characteristics of exosomes make it a challenging carrier in brain targeting of therapeutics and diagnostics. For example, exosomes have long retention within the blood due to high CD47 expression that prevents its phagocytosis via macrophages and monocytes by interacting with SIRPα [201, 204]
3. Loading: It is done by endogenous techniques like natural biogenesis and exogenous techniques as mentioned in section loading of exosomes. Large loads lead to exosomes aggregation and thus its instability. Furthermore, loading efficiency is also variable in nature [76, 201].
4. Production: production of exosomes on large scale is one of the major requirements for using exosomes in clinical use. Various methods of its production are mentioned in the section “Methods of exosomes isolation and purification”. Along with its scalability, integrity, functionality and purity are an important consideration for therapy [20, 205–207].
5. Hypoxic cellular conditions: Hypoxia is a severe cellular stress that regulates exosome release and consequently alters their contents and hence it have been studied in several types of hypoxia disorders, reflecting their biological origin and disease status via bioactive cargo, making exosomes helpful as a possible biomarker for diagnosis or prediction of hypoxic diseases. Exosomes produced during hypoxia, for example, ExoHypoxia, serve a crucial function in assisting cancer cells to cross-talk with microenvironment constituents to establish conditions favourable for cancer growth and metastatic propagation [217, 218].

## Conclusion and future prospective

In the near future, more massive and accurate medicine of exosomal material may be used for the treatment of brain disorders by optimizing exosomal nanocarrier formulation. Much advancement in the field is anticipated in the future, given the positive efforts made to verify exosome potential to treat brain diseases, bring them to clinical trials and the pharmaceutical aspects of it will remain to be explored in more detail in the future. In order to understand exosome biogenesis, cargo selection and release in vivo, more sophisticated techniques and methodologies need to be established. There is also a need to consider awareness of how exosomes distinguish the normal part from the damaged part of the brain. Specific proteins and microRNAs responsible for the neuroprotective properties of exosomes should be established. Exosomes is a complex system that raises the problems of protection and predictability, so future research should concentrate on an exosome mimetic delivery system targeting particular cells and the desired therapeutic molecule. It is harder to envision the use of exosomes as a drug product, and only in minimal, personalized medicine configurations can possible applications be realized. In future research, it is important to determine if exosome therapy has a long-term lasting decrease in chronic deficiency of the brain. Future studies are important in order to better identify different aspects of cerebral-targeted exosomes in small and large animal models and to translate the findings into human clinical applications. The challenging task is the use of exosomes for brain targeting and needs close co-operation from multi-disciplinary fields such as academia, industry, clinicians and regulatory agencies. The different studies reported in this paper suggest that in the future, exosome-based formulations may be useful tools for neurodegenerative disorder therapy. Therefore, as the research continues, exosome-based drug delivery to the brain will have a huge opportunity and broader prospect to cure cerebral disease in the near future.

## Data Availability

This is a review article, and hence, it is based upon the earlier research in the proposed arena.

## Abbreviations

CSF: Cerebrospinal fluid
RNA: Ribonucleic acid
DNA: Deoxyribonucleic acid
DALYs: Disability-adjusted life years
RES: Reticuloendothelial system
ILVs: Intraluminal vesicles
MVBs: Multivesicular bodies
ESCRT: Endosomal sorting complex required for transport
GTPase: Guanosine triphosphate–binding protein
ARF6: ADP-ribosylation factor 6
SNARE: Soluble N-ethylmaleimide-sensitive factor attachment protein receptor complex
FasL: Fas ligand
TRAIL: Tumour necrosis factor–related apoptosis-inducing ligand
mRNAs: Messenger RNA
MHC: Major histocompatibility complex
CD86: Cluster of differentiation 86
EGF: Epidermal growth factor
CD 47: Cluster of differentiation 47
MSC: exo exosomes derived from mesenchymal stem cells
TSG 101: tumour susceptibility gene 101
HSP 70: heat shock protein 70
CD9: cluster of differentiation 9
BNIP Bcl-2: and nineteen kilodalton-interacting proteins 2
ELISA: enzyme-linked immunosorbent assay
EX01-8: exosome isolation kit
PEG: polyethylene glycol
anti-HIV: Anti-human immunodeficiency virus
TLR3: toll-like receptor-3
BDNF: brain-derived neurotropic factor
DEP: dielectrophoretic separation
DLD: deterministic lateral displacement
TEM: transmission electron microscopy
BBB: blood-brain barrier
BMEC: brain microvascular endothelial cells
LFA-1: integrin lymphocyte function–associated antigen 1
ICAM 1: intercellular adhesion molecule 1
TfR: transferrin receptors
RVG: rabies virus glycoprotein
RGE: arginine-glycine-glutamic acid
RGD: arginyl-glycyl-aspartic acid
VEGF: vascular endothelial growth factor
bEND: brain endothelium
siRNA: small-interfering RNA
VEGF: vascular endothelial growth factor
PET: positron emissions tomography
GM 3: monosialodihexosylganglioside
TSG 101: tumour susceptibility gene 101
SPIONs: superparamagnetic iron oxide nanoparticles
tPA: tissue plasminogen activator
AIS: acute Ischemic Stroke
PCR: polymerase chain reaction
IS: ischemic stroke
HIS: hyper-acute phase IS
AIS: acute ischemic stroke
SIS: subacute phase IS
RIS: recovery phase IS
MSC: mesenchymal stem cell
MCAO: middle cerebral artery occlusion
ADSC: adipose-derived stem cell
HIE: hypoxic-ischemic encephalopathy
TBI: traumatic brain injuries
US: United States
ICP: intracranial pressure
SYNPO: synaptopodin
MAP: microtubule associated proteins
p-tau: phosphorylated tau proteins
mTBI: mild TBI
rTBI: repetitive TBI
NfL: neurofilament light chain
TNF-α: tumour necrosis factor-α
IL-6 and IL-10: interleukins 6 and 10
PCS: post-concussive syndrome
PTSD: post-traumatic stress disorder
CCI: controlled cortical impact
F9: Christmas factor
THBS1: thrombospondin-1
APP: myloid-β precursor protein
mTLE-HS: mesial temporal lobe epilepsy related to hippocampal sclerosis
GGE: genetic generalized epilepsies
RVG: rabies virus glycoprotein
MOR: opioid receptor Mu
CPP: conditioned place preference
mTOR: mechanistic target of rapamycin
PCA3: prostate cancer antigen 3
qRT-PCR: quantitative reverse transcriptase-polymerase chain reaction
lncRNAs: long non-coding RNAs
IGLV1-33: protein immunoglobulin lambda variable 1-33
C1q: complement component 1q
FGF2: fibroblast growth factor-2
Rab: Ras-associated binding
NPTX2: neuronal pentraxin 2
NRXN2a: neurexin 2a
AMPA4: GluA4-containing glutamate
NLGN1: neuroligin 1
AKT: serine threonine kinase
BACE1: beta-site amyloid precursor protein cleaving enzyme 1
CNS: central nervous system
ALS: amyotrophic lateral sclerosis
NIR: novel INHAT repressor
HD: Huntington’s disease
CAG: cytosine, adenine and guanine
REST: RE1-silencing transcription factor
GBM: glioblastoma
MAPK: mitogen-activated protein kinase
EGFRvIII: epidermal growth factor recipient variant III
pre-DGU: density gradient ultracentrifugation
HFE: human homeostatic iron regulator
IDHMUT: isocitrate dehydrogenase mutation
DC: dendritic cells
DEX: dendritic cell–derived exosomes
MHC: major histocompatibility complex
Lamp2b: lysosome-associated membrane protein 2
NK: natural killer
cRGD: curcumin-loaded arginyl-glycyl-aspartic acid
EGFR: epidermal growth factor receptor
LPS: lipopolysaccharides
ADEs: astrocyte-derived exosomes
CPs: complement protein levels
MDD: major depressive disorder
AE: autoimmune encephalitis
GTPase: guanylate-binding protein-1
BM: bacterial meningitis
GMP: good manufacturing practice
GLP: good laboratory practice
GDP: good documentary practice
GDC: good clinical practice
GSP: Good storage practice
SIRPα: signal regulatory protein α

## References

[1] GBD. Neurology Collaborators 2019. Global, regional, and national burden of neurological disorders, 1990–2016: a systematic analysis for the Global Burden of Disease Study. Lancet Neurol. 2016;18(5):459–480. 10.1016/S1474-4422(18)30499-X PMC6459001 30879893

[2] Dang XTT, Kavishka JM, Zhang DX, Pirisinu M, Le MTN (2020). Extracellular vesicles as an efficient and versatile system for drug delivery. Cells.

[3] Rousseau M, Belleannee C, Duchez AC, Cloutier N, Levesque T, Jacques F, et al. Detection and quantification of microparticles from different cellular lineages using flow cytometry. Evaluation of the impact of secreted phospholipase A2 on microparticle assessment. PLOS ONE. 2015;10(1):e0116812. 10.1371/journal.pone.0116812 PMC4294685 25587983

[4] Meldolesi J (2018). Exosomes and ectosomes in intercellular communication. Curr Biol.

[5] Vlaeminck-Guillem V. Extracellular vesicles in prostate cancer carcinogenesis, diagnosis, and management. Front Oncol. 2018;8:1–22. 10.3389/fonc.2018.00222 PMC6008571 29951375

[6] Ma L, Li Y, Peng J, Wu D, Zhao X, Cui Y (2014). Discovery of the migrasome, an organelle mediating release of cytoplasmic contents during cell migration. Cell Res.

[7] Ciardiello C, Leone A, Lanuti P, Roca MS, Moccia T, Minciacchi VR, et al. Large oncosomes overexpressing integrin alpha-V promote prostate cancer adhesion and invasion via AKT activation. J Exp Clin Cancer Res. 2019;38(1):317. 10.1186/s13046-019-1317-6 PMC6639931 31319863

[8] Skogberg G, Telemo E, Ekwall O. Exosomes in the thymus: antigen transfer and vesicles. Front Immunol. 2015;6:366. 10.3389/fimmu.2015.00366 PMC4507453 26257734

[9] Campbell LA, Coke LM, Richie CT, Fortuno LV, Park AY, Harvey BK (2019). Gesicle-mediated delivery of CRISPR/Cas9 ribonucleoprotein complex for inactivating the HIV provirus. Mol Ther..

[10] Ailuno G, Baldassari S, Lai F, Florio T, Caviglioli G (2020). Exosomes and extracellular vesicles as emerging theranostic platforms in cancer research. Cells.

[11] Harding C, Stahl P. Transferrin recycling in reticulocytes: pH and iron are important determinants of ligand binding and processing. Biochem Biophys Res Commun. 1983;113:650–8. 10.1016/0006-291x(83)91776-x 6870878

[12] Pan BT, Johnstone RM. Fate of the transferrin receptor during maturation of sheep reticulocytes in vitro: selective externalization of the receptor. Cell. 1983;33:967–78. 10.1016/0092-8674(83)90040-5 6307529

[13] Jung MK, Mun JY. Sample preparation and imaging of exosomes by transmission electron microscopy. J Vis Exp. 2018;131:1–5. 10.3791/56482 PMC5908436 29364263

[14] Zhang L, Yu D. Exosomes in cancer development, metastasis, and immunity. Biochimica et Biophysica Acta (BBA) - Reviews on Cancer. 2019;1871(2):455–468 10.1016/j.bbcan.2019.04.004 PMC6542596 31047959

[15] Smyth T, Kullberg M, Malik N, Smith-Jones P, Graner MW, Anchordoquy TJ. Biodistribution and delivery efficiency of unmodified tumor-derived exosomes. J Control Release. 2015;199:145–55. 10.1016/j.jconrel.2014.12.013 PMC4441346 25523519

[16] Bobrie A, Colombo M, Raposo G, Théry C (2011). Exosome secretion: molecular mechanisms and roles in immune responses. Traffic.

[17] Henne WM, Buchkovich NJ, Emr SD (2011). The ESCRT pathway. Dev Cell.

[18] Tanziela T, Shaikh S, Jiang H, Lu Z, Wang X. Efficient encapsulation of biocompatible nanoparticles in exosomes for cancer theranostics. Nano Today. 2020;35:100964.

[19] Hurley JH (2015). ESCRTs are everywhere. EMBO J.

[20] Böing AN, van der Pol E, Grootemaat AE, Coumans FAW, Sturk A, Nieuwland R (2014). Single-step isolation of extracellular vesicles by size-exclusion chromatography. Journal of Extracellular Vesicles.

[21] Ghossoub R, Lembo F, Rubio A, Gaillard CB, Bouchet J, Vitale N, et al. Syntenin-ALIX exosome biogenesis and budding into multivesicular bodies are controlled by ARF6 and PLD2. Nat Commun. 2014;5:3477. 10.1038/ncomms4477 24637612

[22] Kawikova I, Askenase PW. Diagnostic and therapeutic potentials of exosomes in CNS diseases. Brain Res. 2015;1617:63–71. 10.1016/j.brainres.2014.09.070 PMC4862949 25304360

[23] Lo Cicero A, Stahl PD, Raposo G. Extracellular vesicles shuffling intercellular messages: for good or for bad. Curr Opin Cell Biol. 2015;35:69–77. 10.1016/j.ceb.2015.04.013 26001269

[24] Kang H, Kim J, Park J (2017). Methods to isolate extracellular vesicles for diagnosis. Micro and Nano Systems Letters.

[25] Trajkovic K, Hsu C, Chiantia S, Rajendran L, Wenzel D, Wieland F (2008). Ceramide triggers budding of exosome vesicles into multivesicular endosomes. Science.

[26] Ostrowski M, Carmo NB, Krumeich S, Fanget I, Raposo G, Savina A (2010). Rab27a and Rab27b control different steps of the exosome secretion pathway. Nat Cell Biol.

[27] Hsu C, Morohashi Y, Yoshimura S, Manrique-Hoyos N, Jung S, Lauterbach MA (2010). Regulation of exosome secretion by Rab35 and its GTPase-activating proteins TBC1D10A–C. J Cell Biol.

[28] Baietti MF, Zhang Z, Mortier E, Melchior A, Degeest G, Geeraerts A, Ivarsson Y, Depoortere F, Coomans C, Vermeiren E, Zimmermann P, David G (2012). Syndecan-syntenin-ALIX regulates the biogenesis of exosomes. Nat Cell Biol..

[29] Gu H, Chen C, Hao X, Wang C, Zhang X, Li Z (2016). Sorting protein VPS33B regulates exosomal autocrine signaling to mediate hematopoiesis and leukemogenesis. J Clin Investig.

[30] Fader CM, Sánchez DG, Mestre MB, Colombo MI (2009). TI-VAMP/VAMP7 and VAMP3/cellubrevin: two v-SNARE proteins involved in specific steps of the autophagy/multivesicular body pathways. Biochim Biophys Acta.

[31] Parolini I, Federici C, Raggi C, Lugini L, Palleschi S, De Milito A (2009). Microenvironmental pH is a key factor for exosome traffic in tumor cells. J Biol Chem.

[32] Zylbersztejn K, Galli T (2011). Vesicular traffic in cell navigation. FEBS J.

[33] Zhang Y, Liu Y, Liu H, Tang WH (2019). Exosomes: biogenesis, biologic function and clinical potential. Cell Biosci.

[34] Alvarez-Erviti L, Seow Y, Yin H, Betts C, Lakhal S, Wood MJ (2011). Delivery of siRNA to the mouse brain by systemic injection of targeted exosomes. Nat Biotechnol.

[35] Qu M, Lin Q, Huang L, Fu Y, Wang L, He S, et al. Dopamine-loaded blood exosomes targeted to brain for better treatment of Parkinson’s disease. J Control Release. 2018;287:156–66. 10.1016/j.jconrel.2018.08.035 30165139

[36] Tian T, Zhang HX, He CP, Fan S, Zhu YL, Qi C, et al. Surface functionalized exosomes as targeted drug delivery vehicles for cerebral ischemia therapy. Biomaterials. 2018;150:137–49. 10.1016/j.biomaterials.2017.10.012 29040874

[37] Wood MJ, O’Loughlin AJ, Samira L (2011). Exosomes and the blood-brain barrier: implications for neurological diseases. Ther Deliv.

[38] Ha D, Yang N, Nadithe V (2016). Exosomes as therapeutic drug carriers and delivery vehicles across biological membranes: current perspectives and future challenges. Acta Pharmaceutica Sinica B.

[39] Khongkow M, Yata T, Boonrungsiman S, Ruktanonchai UR, Graham D, Namdee K (2019). Surface modification of gold nanoparticles with neuron-targeted exosome for enhanced blood–brain barrier penetration. Sci Rep.

[40] Liu Y, Li D, Liu Z, Zhou Y, Chu D, Li X (2015). Targeted exosome-mediated delivery of opioid receptor Mu siRNA for the treatment of morphine relapse. Sci Rep.

[41] Simpson RJ, Jensen SS, Lim JW (2008). Proteomic profiling of exosomes: current perspectives. Proteomics.

[42] Skog J, Würdinger T, van Rijn S, Meijer DH, Gainche L, Sena-Esteves M (2008). Glioblastoma microvesicles transport RNA and proteins that promote tumour growth and provide diagnostic biomarkers. Nat Cell Biol.

[43] El-Andaloussi S, Lee Y, Lakhal-Littleton S, Li J, Seow Y, Gardiner C (2013). Exosome-mediated delivery of siRNA in vitro and in vivo. Nat Protoc.

[44] Ribeiro MF, Zhu H, Millard RW, Fan GC (2013). Exosomes function in pro- and anti-angiogenesis. Curr Angiogenes.

[45] Mehryab F, Rabbani S, Shahhosseini S, Shekari F, Fatahi Y, Baharvand H, et al. Exosomes as a next-generation drug delivery system: an update on drug loading approaches, characterization, and clinical application challenges. Acta Biomater. 2020;113:42–62. 10.1016/j.actbio.2020.06.036 32622055

[46] Yang T, Martin P, Fogarty B, Brown A, Schurman K, Phipps R (2015). Exosome delivered anticancer drugs across the blood-brain barrier for brain cancer therapy in Danio rerio. Pharm Res.

[47] Jia G, Han Y, An Y, Ding Y, He C, Wang X, et al. NRP-1 targeted and cargo-loaded exosomes facilitate simultaneous imaging and therapy of glioma in vitro and in vivo. Biomaterials. 2018;178:302–16. 10.1016/j.biomaterials.2018.06.029 29982104

[48] Kim G, Kim M, Lee Y, Byun JW, Hwang DW, Lee M. Systemic delivery of microRNA-21 antisense oligonucleotides to the brain using T7-peptide decorated exosomes. J Control Release. 2020;317:273–81. 10.1016/j.jconrel.2019.11.009 31730913

[49] Rufino-Ramos D, Albuquerque PR, Carmona V, Perfeito R, Nobre RJ, Pereira de Almeida L. Extracellular vesicles: novel promising delivery systems for therapy of brain diseases. J Control Release. 2017;262:247–58. 10.1016/j.jconrel.2017.07.001 28687495

[50] Tauro BJ, Greening DW, Mathias RA, Ji H, Mathivanan S, Scott AM (2012). Comparison of ultracentrifugation, density gradient separation, and immunoaffinity capture methods for isolating human colon cancer cell line LIM1863-derived exosomes. Methods.

[51] Batrakova EV, Kim MS. Using exosomes, naturally-equipped nanocarriers, for drug delivery. J Control Release. 2015;219:396–405. 10.1016/j.jconrel.2015.07.030 PMC4656109 26241750

[52] Du L, Jiang Y, Sun Y. Astrocyte-derived exosomes carry microRNA-17-5p to protect neonatal rats from hypoxic-ischemic brain damage via inhibiting BNIP-2 expression. Neurotoxicology. 2020;83:28–39. 10.1016/j.neuro.2020.12.006 33309839

[53] He R, Jiang Y, Shi Y, Liang J, Zhao L. Curcumin-laden exosomes target ischemic brain tissue and alleviate cerebral ischemia-reperfusion injury by inhibiting ROS-mediated mitochondrial apoptosis. Mater Sci Eng C Mater Biol Appl. 2020;117:111314. 10.1016/j.msec.2020.111314 32919674

[54] Dash M, Palaniyandi K, Ramalingam S, Sahabudeen S, Raja NS. Exosomes isolated from two different cell lines using three different isolation techniques show variation in physical and molecular characteristics. Biochim Biophys Acta Biomembr. 2021;1863(2):183490. 10.1016/j.bbamem.2020.183490 33212036

[55] Bullock KM, Sharma P, Whiteside TL, Banks WA. Abstract # 2057 Inflammation and blood-to-brain transport of tumor-derived exosomes. Brain Behav Immun. 2019;76:e5.

[56] Soraya H, Sani NA, Jabbari N, Rezaie J (2020). Metformin increases exosome biogenesis and secretion in U87 MG human glioblastoma cells: a possible mechanism of therapeutic resistance. Arch Med Res.

[57] Sun L, Wang X, Zhou Y, Zhou RH, Ho WZ, Li JL. Exosomes contribute to the transmission of anti-HIV activity from TLR3-activated brain microvascular endothelial cells to macrophages. Antiviral Res. 2016;134:167–71. 10.1016/j.antiviral.2016.07.013 PMC5310846 27496004

[58] Fang K, Xu JX, Chen XX, Gao XR, Huang LL, Du AQ, et al. Differential serum exosome microRNA profile in a stress-induced depression rat model. J Affect Disord. 2020;274:144–58. 10.1016/j.jad.2020.05.017 32469797

[59] Rani K, Rastogi S, Vishwakarma P, Bharti PS, Sharma V, Renu KM, et al. A novel approach to correlate the salivary exosomes and their protein cargo in the progression of cognitive impairment into Alzheimer’s disease. J Neurosci Methods. 2021;347:108980. 10.1016/j.jneumeth.2020.108980 33075328

[60] Patil SM, Sawant SS, Kunda NK. Exosomes as drug delivery systems: a brief overview and progress update. Eur J Pharm Biopharm. 2020;154:259–69. 10.1016/j.ejpb.2020.07.026 32717385

[61] Yuan D, Zhao Y, Banks WA, Bullock KM, Haney M, Batrakova E, Kabanov AV. Macrophage exosomes as natural nanocarriers for protein delivery to inflamed brain. Biomaterials. 2017;142:1–12. 10.1016/j.biomaterials.2017.07.011 PMC5603188 28715655

[62] Willingham SB, Volkmer JP, Gentles AJ, Sahoo D, Dalerba P, Mitra SS (2012). The CD47-signal regulatory protein alpha (SIRPa) interaction is a therapeutic target for human solid tumors. Proc Natl Acad Sci U S A.

[63] Lou G, Song X, Yang F, Wu S, Wang J, Chen Z, Liu Y. Exosomes derived from miR-122-modified adipose tissue-derived MSCs increase chemosensitivity of hepatocellular carcinoma. J Hematol Oncol. 2015;8:122. 10.1186/s13045-015-0220-7 PMC4627430 26514126

[64] Darband SG, Mirza-Aghazadeh-Attari M, Kaviani M, Mihanfar A, Sadighparvar S, Yousefi B. Exosomes: natural nanoparticles as bio shuttles for RNAi delivery. J Control Release. 2018;289:158–70. 10.1016/j.jconrel.2018.10.001 30290245

[65] Reshke R, Taylor JA, Savard A, Guo H, Rhym LH, Kowalski PS (2020). Reduction of the therapeutic dose of silencing RNA by packaging it in extracellular vesicles via a pre-microRNA backbone. Nat Biomed Eng.

[66] Yang J, Wu S, Hou L, Zhu D, Yin S, Yang G, et al. Therapeutic effects of simultaneous delivery of nerve growth factor mRNA and protein via exosomes on cerebral ischemia. Mol Ther Nucleic Acids. 2020;21:512–22. 10.1016/j.omtn.2020.06.013 PMC7365960 32682291

[67] Keppler OT, Stehling P, Herrmann M, Kayser H, Grunow D, Reutter W, Pawlita M (1995). Biosynthetic modulation of sialic acid-dependent virus-receptor interactions of two primate polyoma viruses. J Biol Chem.

[68] Du J, Che PL, Wang ZY, Aich U, Yarema KJ (2011). Designing a binding interface for control of cancer cell adhesion via 3D topography and metabolic oligosaccharide engineering. Biomaterials.

[69] Krauss Juillerat F, Borcard F, Staedler D, Scaletta C, Applegate LA, Comas H (2012). Functionalization of microstructured open-porous bioceramic scaffolds with human fetal bone cells. Bioconjug Chem.

[70] Sun D, Zhuang X, Xiang X, Liu Y, Zhang S, Liu C (2010). A novel nanoparticle drug delivery system: the anti-inflammatory activity of curcumin is enhanced when encapsulated in exosomes. Mol Ther.

[71] Zhuang X, Xiang X, Grizzle W, Sun D, Zhang S, Axtell RC (2011). Treatment of brain inflammatory diseases by delivering exosome encapsulated anti-inflammatory drugs from the nasal region to the brain. Mol Ther.

[72] Tang K, Zhang Y, Zhang H, Xu P, Liu J, Ma J, et al. Delivery of chemotherapeutic drugs in tumour cell-derived microparticles. Nat Commun. 2012;3:1282. 10.1038/ncomms2282 23250412

[73] Fuhrmann G, Serio A, Mazo M, Nair R, Stevens MM. Active loading into extracellular vesicles significantly improves the cellular uptake and photodynamic effect of porphyrins. J Control Release. 2015;205:35–44. 10.1016/j.jconrel.2014.11.029 25483424

[74] Aqil F, Kausar H, Agrawal AK, Jeyabalan J, Kyakulaga AH, Munagala R, Gupta R (2016). Exosomal formulation enhances therapeutic response of celastrol against lung cancer. Exp Mol Pathol.

[75] Qi H, Liu C, Long L, Ren Y, Zhang S, Chang X, Qian X, Jia H, Zhao J, Sun J, Hou X, Yuan X, Kang C (2016). Blood exosomes endowed with magnetic and targeting properties for cancer therapy. ACS Nano.

[76] Wahlgren J, De L Karlson T, Brisslert M, Vaziri Sani F, Telemo E, Sunnerhagen P. Plasma exosomes can deliver exogenous short interfering RNA to monocytes and lymphocytes. Nucleic Acids Res. 2012;40(17):e130. 10.1093/nar/gks463 PMC3458529 22618874

[77] Cooper JM, Wiklander PB, Nordin JZ, Al-Shawi R, Wood MJ, Vithlani M (2014). Systemic exosomal siRNA delivery reduced alpha-synuclein aggregates in brains of transgenic mice. Mov Disord.

[78] Hood JL, Scott MJ, Wickline SA. Maximizing exosome colloidal stability following electroporation. Anal Biochem. 2014;448:41–9. 10.1016/j.ab.2013.12.001 PMC3954633 24333249

[79] Li T, Yan Y, Wang B, Qian H, Zhang X, Shen L (2013). Exosomes derived from human umbilical cord mesenchymal stem cells alleviate liver fibrosis. Stem Cells Dev.

[80] Podolak I, Galanty A, Sobolewska D (2010). Saponins as cytotoxic agents: a review. Phytochem Rev.

[81] Haney MJ, Klyachko NL, Zhao Y, Gupta R, Plotnikova EG, He Z, et al. Exosomes as drug delivery vehicles for Parkinson’s disease therapy. J Control Release. 2015;207:18–30. 10.1016/j.jconrel.2015.03.033 PMC4430381 25836593

[82] Chen CC, Liu L, Ma F, Wong CW, Guo XE, Chacko JV (2016). Elucidation of exosome migration across the blood-brain barrier model in vitro. Cell Mol Bioeng.

[83] Dong X (2018). Current strategies for brain drug delivery. Theranostics.

[84] Cui GH, Wu J, Mou FF, Xie WH, Wang FB, Wang QL (2018). Exosomes derived from hypoxia-preconditioned mesenchymal stromal cells ameliorate cognitive decline by rescuing synaptic dysfunction and regulating inflammatory responses in APP/PS1 mice. FASEB J.

[85] Zhao C, Wang H, Xiong C, Liu Y (2018). Hypoxic glioblastoma release exosomal VEGF-A induce the permeability of blood-brain barrier. Biochem Biophys Res Commun.

[86] Fan Y, Li Y, Huang S, Xu H, Li H, Liu B. Resveratrol-primed exosomes strongly promote the recovery of motor function in SCI rats by activating autophagy and inhibiting apoptosis via the PI3K signaling pathway. Neurosci Lett. 2020;736:135262. 10.1016/j.neulet.2020.135262 32682847

[87] Alyautdin R, Khalin I, Nafeeza MI, Haron MH, Kuznetsov D. Nanoscale drug delivery systems and the blood-brain barrier. Int J Nanomedicine. 2014;9:795–811. 10.2147/IJN.S52236 PMC3926460 24550672

[88] Record M, Subra C, Silvente-Poirot S, Poirot M (2011). Exosomes as intercellular signalosomes and pharmacological effectors. Biochem Pharmacol.

[89] Zhou W, Fong MY, Min Y, Somlo G, Liu L, Palomares MR (2014). Cancer-secreted miR-105 destroys vascular endothelial barriers to promote metastasis. Cancer Cell.

[90] Tominaga N, Kosaka N, Ono M, Katsuda T, Yoshioka Y, Tamura K. Brain metastatic cancer cells release microRNA-181c-containing extracellular vesicles capable of destructing blood-brain barrier. Nat Commun. 2015;6:6716. 10.1038/ncomms7716 PMC4396394 25828099

[91] Yang T, Fogarty B, LaForge B, Aziz S, Pham T (2017). Delivery of small interfering RNA to inhibit vascular endothelial growth factor in zebrafish using natural brain endothelia cell-secreted exosome nanovesicles for the treatment of brain cancer. AAPS J.

[92] Liu Y, Fu N, Su J, Wang X, Li X. Rapid enkephalin delivery using exosomes to promote neurons recovery in ischemic stroke by inhibiting neuronal p53/caspase-3, Biomed Res Int. 2019;4273290 10.1155/2019/4273290 PMC6425296 30949500

[93] Lin J, Li J, Huang B, Liu J, Chen X, Chen XM, et al. Exosomes: novel biomarkers for clinical diagnosis. Sci World J. 2015;657086. 10.1155/2015/657086 PMC4322857 25695100

[94] Kanninen KM, Bister N, Koistinaho J, Malm T (2016). Exosomes as new diagnostic tools in CNS diseases. Biochim Biophys Acta.

[95] Pusic AD, Pusic KM, Clayton BL, Kraig RP (2014). IFNγ-stimulated dendritic cell exosomes as a potential therapeutic for remyelination. J Neuroimmunol.

[96] Wei H, Xu Y, Xu W, Zhou Q, Chen Q, Yang M, et al. Serum exosomal miR-223 serves as a potential diagnostic and prognostic biomarker for dementia. Neuroscience. 2018;379:167–76. 10.1016/j.neuroscience.2018.03.016 29559383

[97] Yan S, Zhang H, Xie W (2017). Altered microRNA profiles in plasma exosomes from mesial temporal lobe epilepsy with hippocampal sclerosis. Oncotarget.

[98] Wang G, Dinkins M, He Q, Zhu G, Poirier C, Campbell A (2012). Astrocytes secrete exosomes enriched with proapoptotic ceramide and prostate apoptosis response 4 (PAR-4): potential mechanism of apoptosis induction in Alzheimer disease (AD). J Biol Chem.

[99] Willms E, Johansson HJ, Mäger I, Lee Y, Blomberg KE, Sadik M, et al. Cells release subpopulations of exosomes with distinct molecular and biological properties. Sci Rep. 2016;6:22519. 10.1038/srep22519 PMC4773763 26931825

[100] Orefice NS, Souchet B, Braudeau J, Alves S, Piguet F, Collaud F, et al. Real-time monitoring of exosome enveloped -AAV spreading by endomicroscopy approach: a new tool for gene delivery in the brain. Mol Ther Methods Clin Dev. 2019;14:237–51. 10.1016/j.omtm.2019.06.005 PMC6699252 31440523

[101] Kalani A, Chaturvedi P, Kamat PK, Maldonado C, Bauer P, Joshua IG, et al. Curcumin-loaded embryonic stem cell exosomes restored neurovascular unit following ischemia-reperfusion injury. Int J Biochem Cell Biol. 2016;79:360–9. 10.1016/j.biocel.2016.09.002 PMC5067233 27594413

[102] Didiot MC, Hall LM, Coles AH, Haraszti RA, Godinho BM, Chase K (2016). Exosome-mediated delivery of hydrophobically modified siRNA for Huntingtin mRNA silencing. Mol Ther.

[103] Jiang L, Dong H, Cao H, Ji X, Luan S, Liu J. Exosomes in pathogenesis, diagnosis, and treatment of Alzheimer’s disease. Med Sci Monit. 2019;25:3329–35. 10.12659/MSM.914027 PMC6515980 31056537

[104] Ebrahimkhani S, Beadnall HN, Wang C, Suter CM, Barnett MH, Buckland ME (2020). Serum exosome microRNAs predict multiple sclerosis disease activity after fingolimod treatment. Mol Neurobiol.

[105] Chen JJ, Zhao B, Zhao J, Li S. Potential roles of exosomal microRNAs as diagnostic biomarkers and therapeutic application in Alzheimer’s disease. Neural Plast. 2017;2017:7027380. 10.1155/2017/7027380 PMC5523215 28770113

[106] Wu X, Zheng T, Zhang B (2017). Exosomes in Parkinson’s disease. Neurosci Bull.

[107] Chang CL, Chen HH, Chen KH, Chiang JY, Li YC, Lin HS (2019). Adipose-derived mesenchymal stem cell-derived exosomes markedly protected the brain against sepsis syndrome induced injury in rat. Am J Transl Res.

[108] Zhao Y, Haney MJ, Gupta R, Bohnsack JP, He Z, Kabanov AV, et al. GDNF-transfected macrophages produce potent neuroprotective effects in Parkinson’s disease mouse model. PLoS One. 2014;9(9):e106867. 10.1371/journal.pone.0106867 PMC4167552 25229627

[109] Long Q, Upadhya D, Hattiangady B, Kim DK, An SY, Shuai B (2017). Intranasal MSC-derived A1-exosomes ease inflammation, and prevent abnormal neurogenesis and memory dysfunction after status epilepticus. Proc Natl Acad Sci U S A.

[110] Rao P, Benito E, Fischer A. MicroRNAs as biomarkers for CNS disease. Front Mol Neurosci. 2013;6:1–13. 10.3389/fnmol.2013.00039 PMC3840814 24324397

[111] Kumar A, Stoica BA, Loane DJ, Yang M, Abulwerdi G, Khan N (2017). Microglial-derived microparticles mediate neuroinflammation after traumatic brain injury. J Neuroinflammation.

[112] Zhou J, Chen L, Chen B, Huang S, Zeng C, Wu H (2018). Increased serum exosomal miR-134 expression in the acute ischemic stroke patients. BMC Neurol.

[113] Chen Y, Song Y, Huang J, Qu M, Zhang Y, Geng J, et al. Increased circulating exosomal miRNA-223 is associated with acute ischemic stroke. Front Neurol. 2018;8:1–8. 10.3389/fneur.2017.00057 PMC5326773 28289400

[114] Wang W, Li D-B, Li R-Y, Zhou X, Yu D-J, Lan X-Y (2018). Diagnosis of hyperacute and acute ischaemic stroke: the potential utility of exosomal microRNA-21-5p and microRNA-30a-5p. Cerebrovasc Dis.

[115] Phinney DG, Pittenger MF (2017). Concise review: MSC-derived exosomes for cell-free therapy. STEM CELLS.

[116] Doeppner TR, Herz J, Görgens A, Schlechter J, Ludwig A-K, Radtke S (2015). Extracellular vesicles improve post-stroke neuroregeneration and prevent postischemic immunosuppression. Stem Cells Transl Med.

[117] Geng W, Tang H, Luo S, Lv Y, Liang D, Kang X (2019). Exosomes from miRNA-126-modified ADSCs promotes functional recovery after stroke in rats by improving neurogenesis and suppressing microglia activation. American Journal of Translational Research.

[118] Ophelders DRMG, Wolfs TGAM, Jellema RK, Zwanenburg A, Andriessen P, Delhaas T (2016). Mesenchymal stromal cell-derived extracellular vesicles protect the fetal brain after hypoxia-ischemia. Stem Cells Transl Med.

[119] Taylor DD, Gercel-Taylor C. Exosome platform for diagnosis and monitoring of traumatic brain injury. Philosophical Transactions of the Royal Society B: Bio Sci. 2014;369(1652). 10.1098/rstb.2013.0503 PMC4142024 25135964

[120] Kuhlow CJ, Krady JK, Basu A, Levison SW (2003). Astrocytic Ceruloplasmin expression, which is induced by IL-1 and by traumatic brain injury, increases in the absence of the IL-1 Type 1 Receptor. Glia.

[121] Goetzl L, Merabova N, Darbinian N, Martirosyan D, Poletto E, Fugarolas K (2017). Diagnostic potential of neural exosome cargo as biomarkers for acute brain injury. Annals of Clinical and Translational Neurology.

[122] Kenney K, Qu B-X, Lai C, Devoto C, Motamedi V, Walker WC (2018). Higher exosomal phosphorylated tau and total tau among veterans with combat-related repetitive chronic mild Traumatic Brain Injury. Brain Inj.

[123] Guedes VA, Kenney K, Shahim P, Qu B-X, Lai C, Devoto C (2020). Exosomal neurofilament light. Neurology.

[124] Harrison EB, Hochfelder CG, Lamberty BG, Meays BM, Morsey BM, Kelso ML (2016). Traumatic brain injury increases levels of miR-21 in extracellular vesicles: implications for neuroinflammation. FEBS Open Bio.

[125] Ge X-T, Lei P, Wang H-C, Zhang A-L, Han Z-L, Chen X (2014). miR-21 improves the neurological outcome after traumatic brain injury in rats. Sci Rep.

[126] Lin Z, Gu Y, Zhou R, Wang M, Guo Y, Chen Y (2020). Serum exosomal proteins F9 and TSP-1 as potential diagnostic biomarkers for newly diagnosed epilepsy. Front Neurosci.

[127] Wei N, Zhang H, Wang J, Wang S, Lv W, Luo L (2020). The progress in diagnosis and treatment of exosomes and microRNAs on epileptic comorbidity depression. Front Psych.

[128] Martins-Ferreira R, Chaves J, Carvalho C, Bettencourt A, Chorão R, Freitas J (2020). Circulating microRNAs as potential biomarkers for genetic generalized epilepsies: a three-microRNA panel. Eur J Neurol.

[129] Xian P, Hei Y, Wang R, Wang T, Yang J, Li J (2019). Mesenchymal stem cell-derived exosomes as a nanotherapeutic agent for amelioration of inflammation-induced astrocyte alterations in mice. Theranostics.

[130] Liu W, Bai X, Zhang A, Huang J, Xu S, Zhang J (2019). Role of exosomes in central nervous system diseases. Front Mol Neurosci.

[131] Gui Y, Liu H, Zhang L, Lv W, Hu X (2015). Altered microRNA profiles in cerebrospinal fluid exosome in Parkinson disease and Alzheimer disease. Oncotarget.

[132] Cao X, Lu J, Zhao Z, Li M, Lu T, An X, et al. MicroRNA biomarkers of Parkinson’s disease in serum exosome-like microvesicles. Neurosci Lett. 2017;644:94–9. 10.1016/j.neulet.2017.02.045 28223160

[133] Chen H, Liang F, Gu P, Xu B, Xu H, Wang W, et al. Exosomes derived from mesenchymal stem cells repair a Parkinson’s disease model by inducing autophagy. Cell Death Dis. 2020;11:288. 10.1038/s41419-020-2473-5 PMC7184757 32341347

[134] Wang Q, Han C, Wang K, Sui Y, Li Z, Chen N (2019). Integrated analysis of exosomal lncRNA and mRNA expression profiles reveals the involvement of lnc-MKRN2-42:1 in the pathogenesis of Parkinson’s disease. CNS Neurosci Ther.

[135] Jiang C, Hopfner F, Katsikoudi A, Hein R, Catli C, Evetts S (2020). Serum neuronal exosomes predict and differentiate Parkinson’s disease from atypical parkinsonism. J Neurol Neurosurg Psychiatry.

[136] Jiang R, Rong C, Ke R, Meng S, Yan X, Ke H, et al. Differential proteomic analysis of serum exosomes reveals alterations in progression of Parkinson disease. Med. 2019;98(41):e17478. 10.1097/MD.0000000000017478 PMC6799836 31593110

[137] Kumar R, Donakonda S, Müller SA, Bötzel K, Höglinger GU, Koeglsperger T. FGF2 affects Parkinson’s disease-associated molecular networks through exosomal Rab8b/Rab31. Front Genet. 2020;11:572058. 10.3389/fgene.2020.572058 PMC7545478 33101391

[138] Goetzl EJ, Abner EL, Jicha GA, Kapogiannis D, Schwartz JB (2018). Declining levels of functionally specialized synaptic proteins in plasma neuronal exosomes with progression of Alzheimer’s disease. FASEB J.

[139] Cheng L, Vella LJ, Barnham KJ, McLean C, Masters CL, Hill AF. Small RNA fingerprinting of Alzheimer’s disease frontal cortex extracellular vesicles and their comparison with peripheral extracellular vesicles. J Extracell Vesicles. 2020;9:1766822. 10.1080/20013078.2020.1766822 PMC7448944 32922692

[140] Wang H, Sui H, Zheng Y, Jiang Y, Shi Y, Liang J (2019). Curcumin-primed exosomes potently ameliorate cognitive function in AD mice by inhibiting hyperphosphorylation of Tau protein through the AKT/GSK-3β pathway. Nanoscale.

[141] Goetzl EJ, Schwartz JB, Abner EL, Jicha GA, Kapogiannis D. High complement levels in astrocyte-derived exosomes of Alzheimer’s disease. Ann Neurol. 2018;83:544–52. 10.1002/ana.25172 PMC5867263 29406582

[142] Jahangard Y, Monfared H, Moradi A, Zare M, Mirnajafi-Zadeh J, Mowla SJ. Therapeutic effects of transplanted exosomes containing miR-29b to a rat model of Alzheimer’s disease. Front Neurosci. 2020;14:564. 10.3389/fnins.2020.00564 PMC7314926 32625049

[143] Yang L, Zhai Y, Hao Y, Zhu Z, Cheng G (2019). The regulatory functionality of exosomes derived from hUMSCs in 3D culture for Alzheimer’s disease therapy. Small.

[144] Reza-Zaldivar EE, Hernández-Sapiéns MA, Gutiérrez-Mercado YK, Sandoval-Ávila S, Gomez-Pinedo U, Márquez-Aguirre AL (2019). Mesenchymal stem cell-derived exosomes promote neurogenesis and cognitive function recovery in a mouse model of Alzheimer’s disease. Neural Regen Res.

[145] Ding M, Shen Y, Wang P, Xle Z, Xu S, Zhu Z (2018). Exosomes isolated from human umbilical cord mesenchymal stem cells alleviate neuroinflammation and reduce amyloid-beta deposition by modulating microglial activation in Alzheimer’s disease. Neurochem Res.

[146] Perets N, Betzer O, Shapira R, Brenstein S, Angel A, Sadan T, et al. Golden exosomes selectively target brain pathologies in neurodegenerative and neurodevelopmental disorders. Nano Lett. 2019;19:3422–31. 10.1021/acs.nanolett.8b04148 30761901

[147] Hayashi N, Doi H, Kurata Y, Kagawa H, Atobe Y, Funakoshi K, et al. Proteomic analysis of exosome-enriched fractions derived from cerebrospinal fluid of amyotrophic lateral sclerosis patients. Neurosci Res. 2019;160:43–9. 10.1016/j.neures.2019.10.010 31669371

[148] Bonafede R, Turano E, Scambi I, Busato A, Bontempi P, Virla F, et al. ASC-Exosomes ameliorate the disease progression in SODI(G93A) murine model underlining their potential therapeutic use in human ALS. Int J Mol Sci. 2020;21:3651. 10.3390/ijms21103651 PMC7279464 32455791

[149] Chen Y, Xia K, Chen L, Fan D. Increased interleukin-6 levels in the astrocyte-derived exosomes of sporadic amyotrophic lateral sclerosis patients. Front Neurosci. 2019;13:574. 10.3389/fnins.2019.00574 PMC6560167 31231184

[150] Otake K, Kamiguchi H, Hirozane Y (2019). Identification of biomarkers for amyotrophic lateral sclerosis by comprehensive analysis of exosomal mRNAs in human cerebrospinal fluid. BMC Med Genomics.

[151] Lee S, Im W, Ban J, Lee M, Jung K, Lee S (2017). Exosome-based delivery of miR-124 in a Huntington’s disease model. J Mov Disord.

[152] Xu S, Wang J, Ding N, Hu W, Zhang X, Wang B (2015). Exosome-mediated microRNA transfer plays a role in radiation-induced bystander effect. RNA Biol.

[153] Rajagopal C, Harikumar KB. The origin and functions of exosomes in cancer. Front Oncol. 2018;8:66. 10.3389/fonc.2018.00066 PMC5869252 29616188

[154] Au Yeung CL, Co NN, Tsuruga T, Yeung TL, Kwan SY, Leung CS, et al. Exosomal transfer of stroma-derived miR21 confers paclitaxel resistance in ovarian cancer cells through targeting APAF1. Nat Commun. 2016;7:11150. 10.1038/ncomms11150 PMC4820618 27021436

[155] Liao J, Liu R, Shi YJ, Yin LH, Pu YP (2016). Exosome-shuttling microRNA-21 promotes cell migration and invasion-targeting PDCD4 in esophageal cancer. Int J Oncol.

[156] Richards KE, Zeleniak AE, Fishel ML, Wu J, Littlepage LE, Hill R (2017). Cancer-associated fibroblast exosomes regulate survival and proliferation of pancreatic cancer cells. Oncogene.

[157] Maji S, Chaudhary P, Akopova I, Nguyen PM, Hare RJ, Gryczynski I (2017). Exosomal annexin II promotes angiogenesis and breast cancer metastasis. Mol Cancer Res.

[158] Zhang H, Deng T, Liu R, Bai M, Zhou L, Wang X, et al. Exosome-delivered EGFR regulates liver microenvironment to promote gastric cancer liver metastasis. Nat Commun. 2017;8:15016. 10.1038/ncomms15016 PMC5394240 28393839

[159] Al-Nedawi K, Meehan B, Micallef J, Lhotak V, May L, Guha A (2008). Intercellular transfer of the oncogenic receptor EGFRvIII by microvesicles derived from tumour cells. Nat Cell Biol.

[160] Lee TH, Chennakrishnaiah S, Audemard E, Montermini L, Meehan B, Rak J (2014). Oncogenic ras-driven cancer cell vesiculation leads to emission of double-stranded DNA capable of interacting with target cells. Biochem Biophys Res Commun.

[161] Vlassov AV, Magdaleno S, Setterquist R, Conrad R (2012). Exosomes: current knowledge of their composition, biological functions, and diagnostic and therapeutic potentials. Biochim Biophys Acta.

[162] Cumba Garcia LM, Peterson TE, Cepeda MA, Johnson AJ, Parney IF. Isolation and analysis of plasma-derived exosomes in patients with glioma. Front Oncol. 2019;9:651. 10.3389/fonc.2019.00651 PMC6646733 31380286

[163] Whitehead CA, Kaye AH, Drummond KJ, Widodo SS, Mantamadiotis T, Vella LJ (2020). Extracellular vesicles and their role in glioblastoma. Crit Rev Clin Lab Sci.

[164] Indira Chandran V, Welinder C, Månsson AS, Offer S, Freyhult E, Pernemalm M (2019). Ultrasensitive immunoprofiling of plasma extracellular vesicles identifies syndecan-1 as a potential tool for minimally invasive diagnosis of glioma. Clin Cancer Res.

[165] Bronisz A, Wang Y, Nowicki MO, Peruzzi P, Ansari K, Ogawa D (2014). Extracellular vesicles modulate the glioblastoma microenvironment via a tumor suppression signaling network directed by miR-1. Cancer Res.

[166] Gourlay J, Morokoff AP, Luwor RB, Zhu HJ, Kaye AH, Stylli SS. The emergent role of exosomes in glioma. J Clin Neurosci. 2017;35:13–23. 10.1016/j.jocn.2016.09.021 27771233

[167] Mrowczynski OD, Zacharia BE, Connor JR. Exosomes and their implications in central nervous system tumor biology. Prog Neurobiol. 2019;172:71–83. 10.1016/j.pneurobio.2018.06.006 30003942

[168] Ebrahimkhani S, Vafaee F, Hallal S, Wei H, Lee MYT, Young PE, et al. Deep sequencing of circulating exosomal microRNA allows non-invasive glioblastoma diagnosis. NPJ Precis Oncol. 2018;2:28. 10.1038/s41698-018-0071-0 PMC6290767 30564636

[169] Lan F, Qing Q, Pan Q, Hu M, Yu H, Yue X (2018). Serum exosomal miR-301a as a potential diagnostic and prognostic biomarker for human glioma. Cell Oncol (Dordr).

[170] Escudier B, Dorval T, Chaput N, André F, Caby MP, Novault S (2005). Vaccination of metastatic melanoma patients with autologous dendritic cell (DC) derived-exosomes: results of the first phase I clinical trial. J Transl Med.

[171] Katakowski M, Chopp M (2016). Exosomes as tools to suppress primary brain tumor. Cell Mol Neurobiol.

[172] Arscott WT, Tandle AT, Zhao S, Shabason JE, Gordon IK, Schlaff CD (2013). Ionizing radiation and glioblastoma exosomes: implications in tumor biology and cell migration. Transl Oncol.

[173] Luan X, Sansanaphongpricha K, Myers I, Chen H, Yuan H, Sun D (2017). Engineering exosomes as refined biological nanoplatforms for drug delivery. Acta Pharmacol Sin.

[174] Ohno S, Takanashi M, Sudo K, Ueda S, Ishikawa A, Matsuyama N (2013). Systemically injected exosomes targeted to EGFR deliver antitumor microRNA to breast cancer cells. Molecular therapy: The journal of the American Society of Gene Therapy.

[175] Katakowski M, Buller B, Zheng X, Lu Y, Rogers T, Osobamiro O (2013). Exosomes from marrow stromal cells expressing miR-146b inhibit glioma growth. Cancer Lett.

[176] Salarpour S, Forootanfar H, Pournamdari M, Ahmadi-Zeidabadi M, Esmaeeli M, Pardakhty A (2019). Paclitaxel incorporated exosomes derived from glioblastoma cells: comparative study of two loading techniques. Daru.

[177] Zhu Q, Ling X, Yang Y, Zhang J, Li Q, Niu X, et al. Embryonic stem cells-derived exosomes endowed with targeting properties as chemotherapeutics delivery vehicles for glioblastoma therapy. Adv Sci. (Weinheim, Baden-Wurttemberg, Germany). 2019;6(6):1801899 10.1002/advs.201801899 PMC6425428 30937268

[178] Pascual M, Ibáñez F, Guerri C (2020). Exosomes as mediators of neuron-glia communication in neuroinflammation. Neural Regen Res.

[179] Li JJ, Wang B, Kodali MC, Chen C, Kim E, Patters BJ (2018). In vivo evidence for the contribution of peripheral circulating inflammatory exosomes to neuroinflammation. J Neuroinflammation.

[180] Luarte A, Bátiz LF, Wyneken U, Lafourcade C. Potential therapies by stem cell-derived exosomes in CNS diseases: focusing on the neurogenic niche. Stem Cells Int. 2016;2016:5736059. 10.1155/2016/5736059 PMC4853949 27195011

[181] Gupta A, Pulliam L. Exosomes as mediators of neuroinflammation. J Neuroinflammation. 2014;11:68. 10.1186/1742-2094-11-68 PMC3994210 24694258

[182] Huang S, Ge X, Yu J, Han Z, Yin Z, Li Y (2018). Increased miR-124-3p in microglial exosomes following traumatic brain injury inhibits neuronal inflammation and contributes to neurite outgrowth via their transfer into neurons. FASEB J.

[183] Banigan MG, Kao PF, Kozubek JA, Winslow AR, Medina J, Costa J. Differential expression of exosomal microRNAs in prefrontal cortices of schizophrenia and bipolar disorder patients. PLoS One. 2013;8(1):e48814. 10.1371/journal.pone.0048814 PMC3559697 23382797

[184] Pulliam L, Sun B, Mustapic M, Chawla S, Kapogiannis D (2019). Plasma neuronal exosomes serve as biomarkers of cognitive impairment in HIV infection and Alzheimer’s disease. J Neurovirol.

[185] Winston CN, Goetzl EJ, Schwartz JB, Elahi FM, Rissman RA. Complement protein levels in plasma astrocyte-derived exosomes are abnormal in conversion from mild cognitive impairment to Alzheimer’s disease dementia. Alzheimers Dement (Amst). 2019;11:61–6. 10.1016/j.dadm.2018.11.002 PMC6477776 31032394

[186] Gayen M, Bhomia M, Balakathiresan N, Knollmann-Ritschel B (2020). Exosomal microRNAs released by activated astrocytes as potential neuroinflammatory biomarkers. Int J Mol Sci.

[187] Azimi M, Ghabaee M, Moghadasi AN, Noorbakhsh F, Izad M (2018). Immunomodulatory function of Treg-derived exosomes is impaired in patients with relapsing-remitting multiple sclerosis. Immunol Res.

[188] Pieragostino D, Cicalini I, Lanuti P, Ercolino E, di Ioia M, Zucchelli M (2018). Enhanced release of acid sphingomyelinase-enriched exosomes generates a lipidomics signature in CSF of multiple sclerosis patients. Sci Rep.

[189] Zheng T, Pu J, Chen Y, Guo Z, Pan H, Zhang L (2017). Exosomes secreted from HEK293-APP Swe/Ind cells impair the hippocampal neurogenesis. Neurotox Res.

[190] Luarte A, Cisternas P, Caviedes A, Batiz LF, Lafourcade C, Wyneken U, et al. Astrocytes at the hub of the stress response: potential modulation of neurogenesis by miRNAs in astrocyte-derived exosomes. Stem Cells Int. 2017;2017:1719050. 10.1155/2017/1719050 PMC5610870 29081809

[191] Kuwano N, Kato TA, Mitsuhashi M, Sato-Kasai M, Shimokawa N, Hayakawa K. Neuron-related blood inflammatory markers as an objective evaluation tool for major depressive disorder: an exploratory pilot case-control study. J Affect Disord. 2018;240:88–98. 10.1016/j.jad.2018.07.040 30059939

[192] Couch Y, Akbar N, Roodselaar J, Evans MC, Gardiner C, Sargent I (2017). Circulating endothelial cell-derived extracellular vesicles mediate the acute phase response and sickness behaviour associated with CNS inflammation. Sci Rep.

[193] De Rivero Vaccari JP, Brand F 3^rd^, Adamczak S, Lee SW, Perez-Barcena J, Wang MY, Bullock MR, et al. Exosome-mediated inflammasome signaling after central nervous system injury. J Neurochem. 2016;136 Suppl 1(01):39–48. 10.1111/jnc.13036 PMC4516699 25628216

[194] Gu J, Jin T, Li Z, Chen H, Xia H, Xu X, et al. Exosomes expressing neuronal autoantigens induced immune response in antibody-positive autoimmune encephalitis. Mol Immunol. 2021;131:164–70. 10.1016/j.molimm.2020.12.034 33446390

[195] Naschberger E, Lubeseder-Martellato C, Meyer N, Gessner R, Kremmer E, Gessner A, Stürzl M (2006). Human guanylate binding protein-1 is a secreted GTPase present in increased concentrations in the cerebrospinal fluid of patients with bacterial meningitis. Am J Pathol.

[196] Lakhal S, Wood MJ (2011). Intranasal exosomes for treatment of neuroinflammation? Prospects and limitations. Molecular Therapy: The Journal of the American Society of Gene Therapy.

[197] Chen W, Huang L, Tang O, Wang S, Hu C, Zhang X (2020). Progress on diagnosis and treatment of central nervous system tuberculosis. Radiology of Infectious Diseases.

[198] Ioannides P, Giedzinski E, Limoli CL (2016). Evaluating different routes of extracellular vesicle administration for cranial therapies. J Cancer Metastasis Treat.

[199] Wu J, Wang Y, Li L (2017). Functional significance of exosomes applied in sepsis: a novel approach to therapy. Biochim Biophys Acta.

[200] Bellavia D, Raimondi L, Costa V, De Luca A, Carina V, Maglio M (2018). Engineered exosomes: a new promise for the management of musculoskeletal diseases. Biochim Biophys Acta Gen Subj..

[201] Kim YK, Choi Y, Nam G-H, Kim I-S. Functionalized exosome harbouring bioactive molecules for cancer therapy. Cancer Lett. 2020;489:155–62. 10.1016/j.canlet.2020.05.036 32623071

[202] Zhou J, Tan X, Tan Y, Li Q, Ma J, Wang G (2018). Mesenchymal stem cell derived exosomes in cancer progression, metastasis and drug delivery: a comprehensive review. J Cancer.

[203] Lai RC, Arslan F, Lee MM, Sze NS, Choo A, Chen TS (2010). Exosome secreted by MSC reduces myocardial ischemia/reperfusion injury. Stem Cell Res.

[204] Kamerkar S, LeBleu VS, Sugimoto H, Yang S, Ruivo CF, Melo SA, Lee JJ, Kalluri R (2017). Exosomes facilitate therapeutic targeting of oncogenic KRAS in pancreatic cancer. Nature.

[205] Heinemann ML, Ilmer M, Silva LP, Hawke DH, Recio A, Vorontsova MA, et al. Benchtop isolation and characterization of functional exosomes by sequential filtration. J Chromatogr A. 2014;1371:125–35. 10.1016/j.chroma.2014.10.026 25458527

[206] Nordin JZ, Lee Y, Vader P, Mäger I, Johansson HJ, Heusermann W. Ultrafiltration with size-exclusion liquid chromatography for high yield isolation of extracellular vesicles preserving intact biophysical and functional properties. Nanomedicine: Nanomed- Nanotechnol. 2015;11(4):879–83. 10.1016/j.nano.2015.01.003 25659648

[207] Colao IL, Corteling R, Bracewell D, Wall I (2018). Manufacturing exosomes: a promising therapeutic platform. Trends Mol Med.

[208] Litak J, Mazurek M, Grochowski C, Kamieniak P, Roliński J (2019). PD-L1/PD-1 axis in glioblastoma multiforme. Int J Mol Sci.

[209] Szopa W, Burley TA, Kramer-Marek G, Kaspera W. Diagnostic and therapeutic biomarkers in glioblastoma: current status and future perspectives. Biomed Res Int. 2017;2017:8013575. 10.1155/2017/8013575 PMC5337853 28316990

[210] Sun X, Ma X, Wang J, Zhao Y, Wang Y, Bihl JC (2017). Glioma stem cells-derived exosomes promote the angiogenic ability of endothelial cells through miR-21/VEGF signal. Oncotarget.

[211] Zeng A, Wei Z, Yan W, Yin J, Huang X, Zhou X, et al. Exosomal transfer of miR-151a enhances chemosensitivity to temozolomide in drug-resistant glioblastoma. Cancer Lett. 2018;436:10–21. 10.1016/j.canlet.2018.08.004 30102952

[212] Uribe D, Torres Á, Rocha JD, Niechi I, Oyarzún C, Sobrevia L, et al. Multidrug resistance in glioblastoma stem-like cells: role of the hypoxic microenvironment and adenosine signaling. Mol Aspects Med. 2017;55:140–51. 10.1016/j.mam.2017.01.009 28223127

[213] Mallawaaratchy DM, Hallal S, Russell B, Ly L, Ebrahimkhani S, Wei H, et al. Comprehensive proteome profiling of glioblastoma-derived extracellular vesicles identifies markers for more aggressive disease. J Neurooncol. 131(2):233–44. 10.1007/s11060-016-2298-3 PMC5306193 27770278

[214] Kojima R, Bojar D, Rizzi G, Hamri GC, El-Baba MD, Saxena P (2018). Designer exosomes produced by implanted cells intracerebrally deliver therapeutic cargo for Parkinson’s disease treatment. Nat Commun.

[215] Thomi G, Surbek D, Haesler V, Joerger-Messerli M, Schoeberlein A (2019). Exosomes derived from umbilical cord mesenchymal stem cells reduce microglia-mediated neuroinflammation in perinatal brain injury. Stem Cell Res Ther.

[216] Gao G, Li C, Zhu J, Wang Y, Huang Y, Zhao S, et al. Glutaminase 1 regulates neuroinflammation after cerebral ischemia through enhancing microglial activation and pro-inflammatory exosome release. Front Immunol. 2020;11:161. 10.3389/fimmu.2020.00161 PMC7020613 32117296

[217] Duan P, Tan J, Miao Y, Zhang Q (2019). Potential role of exosomes in the pathophysiology, diagnosis, and treatment of hypoxic diseases. Am J Transl Res.

[218] Kumar A, Deep G. Exosomes in hypoxia-induced remodeling of the tumor microenvironment. Cancer Lett. 2020;488:1–8. 10.1016/j.canlet.2020.05.018 32473240

